# Zwitterion‐Lubricated Hydrogel Microspheres Encapsulated with Metformin Ameliorate Age‐Associated Osteoarthritis

**DOI:** 10.1002/advs.202402477

**Published:** 2024-06-14

**Authors:** Jiahui Hou, Yanpeng Lin, Chencheng Zhu, Yupeng Chen, Rongmin Lin, Hancheng Lin, Dahai Liu, Daogang Guan, Bin Yu, Jun Wang, Hangtian Wu, Zhuang Cui

**Affiliations:** ^1^ Devision of Orthopaedics and Traumatology Department of Orthopaedics Nanfang Hospital Southern Medical University Guangzhou Guangdong 510515 China; ^2^ Guangdong Provincial Key Laboratory of Bone and Cartilage Regeneration Medicine Nanfang Hospital Southern Medical University Guangzhou Guangdong 510515 China; ^3^ Department of Radiology Nanfang Hospital Southern Medical University Guangzhou Guangdong 510515 China; ^4^ Department of Biochemistry and Molecular Biology School of Basic Medical Sciences Southern Medical University Guangzhou Guangdong 510515 China; ^5^ Guangdong Provincial Key Laboratory of Single Cell Technology and Application Southern Medical University Guangzhou Guangdong 510515 China; ^6^ School of Medicine Foshan University Foshan Guangdong 528000 China

**Keywords:** age‐related osteoarthritis, cellular senescence, iNOS, lubrication, metformin

## Abstract

Chondrocyte senescence and reduced lubrication play pivotal roles in the pathogenesis of age‐related osteoarthritis (OA). In the present study, highly lubricated and drug‐loaded hydrogel microspheres are designed and fabricated through the radical polymerization of sulfobetaine (SB)‐modified hyaluronic acid methacrylate using microfluidic technology. The copolymer contains a large number of SB and carboxyl groups that can provide a high degree of lubrication through hydration and form electrostatic loading interactions with metformin (Met@SBHA), producing a high drug load for anti‐chondrocyte senescence. Mechanical, tribological, and drug release analyses demonstrated enhanced lubricative properties and prolonged drug dissemination of the Met@SBHA microspheres. RNA sequencing (RNA‐seq) analysis, network pharmacology, and in vitro assays revealed the extraordinary capacity of Met@SBHA to combat chondrocyte senescence. Additionally, inducible nitric oxide synthase (iNOS) has been identified as a promising protein modulated by Met in senescent chondrocytes, thereby exerting a significant influence on the iNOS/ONOO‐/P53 pathway. Notably, the intra‐articular administration of Met@SBHA in aged mice ameliorated cartilage senescence and OA pathogenesis. Based on the findings of this study, Met@SBHA emerges as an innovative and promising strategy in tackling age‐related OA serving the dual function of enhancing joint lubrication and mitigating cartilage senescence.

## Graphical abstract

The functionalized and lubricated microspheres presented in this study hold great promise as a potential approach to attenuate the degenerative alterations associated with age‐related osteoarthritis.

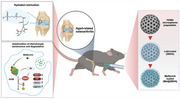

## 1 Introduction

Osteoarthritis (OA) is an age‐associated degenerative joint disorder, leading to debilitating pain and function loss in older adults.^[^ ^1^, ^2^ ^]^ Age is believed to be the most prevalent risk factor for OA pathogenesis, although other risk factors, such as joint injury, obesity, and genetics, have all been associated with the onset of OA.^[^ ^3^ ^]^ Chondrocytes are a unique cell type in articular cartilage. Thus, the functional metabolism in chondrocytes is strongly correlated with articular cartilage homeostasis.^[^ ^4^ ^]^ Cellular senescence is a crucial feature of aging, and compelling evidence demonstrates senescent chondrocytes are significantly elevated in age‐associated OA cartilage.^[^ ^5^, ^6^ ^]^ Senescent cells exhibit growth arrest and senescence‐associated secretory phenotype (SASP), contributing to cartilage disruption and degeneration.^[^ ^7^, ^8^ ^]^ Notably, clearance of senescent cells harbors the capacity to alleviates OA pathogenesis and promotes a regenerative environment.^[^ ^9^, ^10^ ^]^ In summary, chondrocyte senescence is associated with age‐related OA.

Gradual depletion of lubrication is intricately linked to age‐related OA, triggering joint inflammation and subsequent cartilage deterioration.^[^ ^11^ ^]^ Normally, synovial fluid comprises a diverse array of biomacromolecules that serve as highly effective lubricants within the joint under physiological pressure.^[^ ^12^ ^]^ However, in pathological states associated with aging, there is a decline in the concentration of lubricants within the joint,^[^ ^13^ ^]^ which leads to an increase in intra‐articular friction and wear. Therefore, it is imperative to achieve adequate lubrication within the joint to facilitate proper joint mobility and prevent the onset of degenerative alterations.

It is reasonable and desirable to develop an innovative therapeutic intervention that achieves the dual objectives of mitigating chondrocyte senescence and enhancing joint lubrication in age‐related OA. In recent years, significant progress has been made in the development of injectable hydrogels for tissue engineering that exhibit exceptional biocompatibility and controlled drug delivery capabilities in tissue engineering.^[^ ^14^, ^15^ ^]^ However, the limited lubrication capacity of hydrogels has been considered when considered for their clinical application in the treatment of OA. Notably, compared to traditional hydrogels, hydrogel microspheres exhibit notable improvements in flowability and flexibility, facilitating their seamless dispersion and consequent augmentation of lubrication.^[^ ^16^, ^17^ ^]^ The excellent physical rolling ability imparted by the micron‐sized spherical structure of hydrogel microspheres enhances their mobility and local tissue dispersion, allowing them to easily reach deep pathological tissues through syringe needles and perfectly fill any irregular tissue cavities.^[^ ^18^ ^]^ Zwitterionic surfaces have garnered increasing attention because of their significant efficacy in mitigating interfacial friction and augmenting lubricity.^[^ ^19^ ^]^ Zwitterionic polymers, also known as polymers possessing ampholytic attributes, are distinguished by the presence of paired countercharged functional groups within their repeating units.^[^ ^20^ ^]^ When these opposing charges are equitably dispersed within the molecular framework, the polymers exhibit overall charge neutrality and an extraordinary hydration effect through ionic solvation.^[^ ^21^, ^22^ ^]^ Consequently, this enables consistent surface lubrication. Additionally, Recent reports have highlighted the astounding efficacy of metformin (Met) in mitigating the primary manifestations of biological senescence, positioning it as an exceptionally compelling anti‐aging agent.^[^ ^23^ ^]^ However, the investigation of the potential effects of metformin‐encapsulated lubricated hydrogel microspheres on age‐associated OA remains unclear.

**Figure 1:**
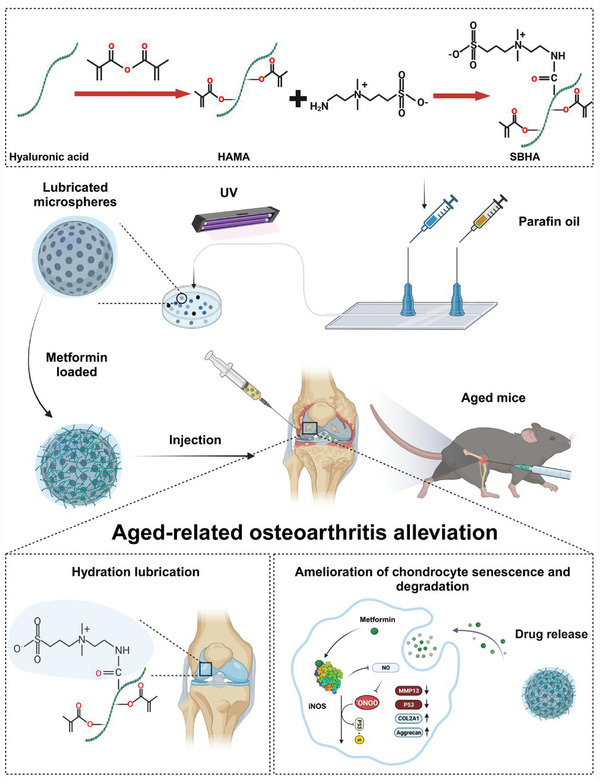
The principle and fabrication of Met@SBHA. A) Synthesis process of SBHA. B) Preparation of Met@SBHA by microfluidic device and photopolymerization process and intra‐articular injection. C) Mechanistic map of the mechanism of alleviating chondrocyte senescence and increasing hydrated lubrication to ameliorate age‐related osteoarthritis. The schematic illustration was created by BioRender.com.

To address these challenges effectively, we propose an innovative biomimetic approach in our current study involving the synthesis of injectable hydrogel microspheres that exhibit improved lubrication properties and allow for the controlled release of Met for OA treatment. As shown in (**Figure** **1**A), using hyaluronic acid methacrylate (HAMA) for the hydrogel microspheres, we chemically conjugated a zwitterionic group of sulfobetaine (SB) and fabricated highly lubricious drug‐loaded hydrogel microspheres (SBHA) guided by microfluidic technology. Subsequently, SBHA was encapsulated with the anti‐senescence drug Met (Met@SBHA) by electrostatic loading interactions and intra‐particularly administered to the knee joints of aged mice to evaluate its therapeutic efficacy (Figure 1B). RNA sequencing (RNA‐seq), network pharmacology, and in vitro and vivo assays were used to elucidate the therapeutic efficacy and underlying mechanisms of Met@SBHA in mitigating age‐related OA (Figure 1C). Met@SBHA devised within the scope of this investigation potentially offers a straightforward and promising strategy for ameliorating age‐related OA, harboring the dual role of augmenting joint lubrication and alleviating cartilage senescence.

## 2 Results

### 2.1 Preparation and Characterization of Met@SBHA

**Figure 2:**
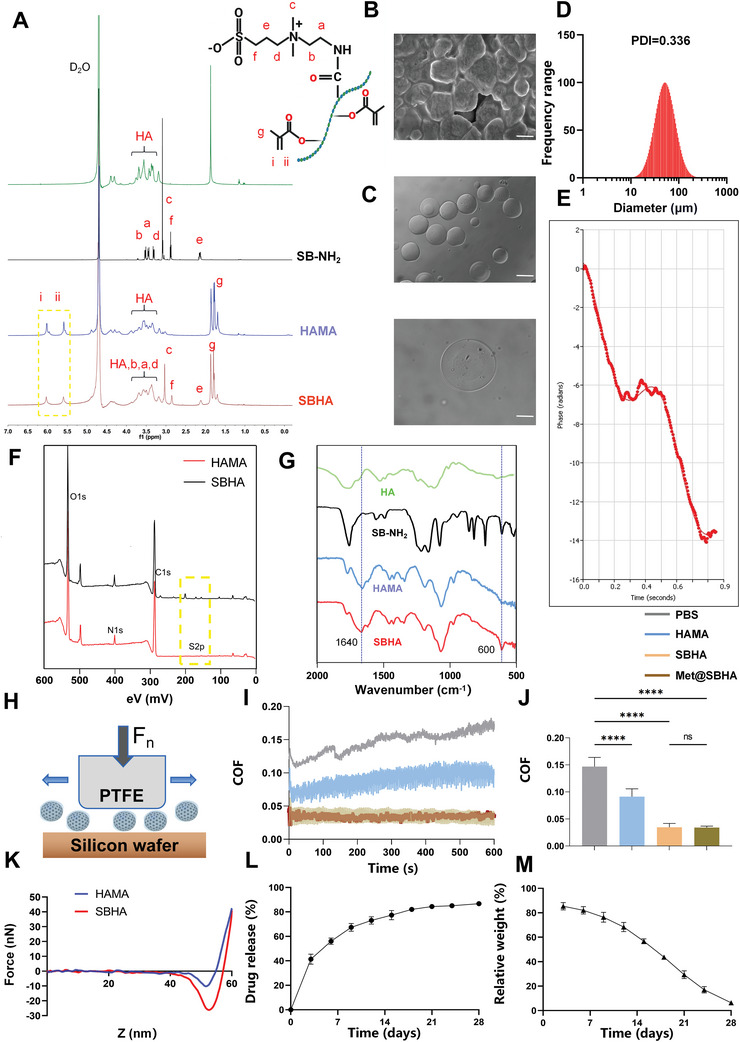
Preparation and characterization of Met@SBHA hydrogel microspheres. A) ^1^H NMR spectrum of hydrogel microspheres composition. B) Electron microscopy of hydrogel microspheres. Scale bars: 500 nm. C) Bright‐field plots of dispersed (Scale bars: 500 µm) and individual hydrogel microspheres (Scale bars: 25 µm) under optical microscope. D) Particle size distributions of hydrogel microspheres. E) Phase analysis light scattering pattern for the analysis of zeta potential. F) X‐ray photoelectron spectroscopy (XPS) of HAMA and SBHA. G) Fourier Transform Infrared Spectroscopy (FTIR) spectroscopy of HA, SB‐NH_2_, HAMA SBHA. H) Friction test diagram. I) COF‐time curves. J) COF histograms for PBS, HAMA, SBHA and Met@SBHA. K) Force‐displacement curves for HAMA and SBHA. L) Drug release profile of Met@SBHA. M) Degradation curve of Met@SBHA. Number of repetitions of all experiments n ≥ 3. Data are presented as the mean ± SD. (*P < 0.05; **P < 0.01; ***P < 0.001 and ****P < 0.0001).

Hyaluronic acid (HA), a typical linear polysaccharide, has been recommended in several clinical guidelines for the management of OA because of its potential to alleviate degenerative processes.^[^ ^24^ ^]^ First, HA is subjected to methacrylic anhydride modification to confer upon HA the property of photocurability, thereby creating HAMA. Successful grafting of the methacrylate groups onto HA was confirmed using ^1^H NMR spectroscopy. The degree of HA methacrylation was 33% (**Figure** **2**A). Subsequently, the zwitterionic group SB‐NH_2_ was used to formulate lubrication agents, facilitating effective boundary lubrication under physiologically elevated pressures. Figure 2A shows the ^1^H NMR spectrum of HAMA‐SB (SBHA). The grafting rate of the SB moiety onto HAMA was quantified by NMR integration at 20%. The characteristic peak of SB‐NH_2_ was detected at approximately 2.2 ppm, which was also observed for SBHA. These results provide conclusive evidence for the successful synthesis of SBHA copolymers. SBHA microspheres were created through the photopolymerization of SBHA droplets using microfluidic technology. The hydrogel microspheres precipitated after standing in solution and formed a uniformly dispersed semi‐transparent suspension after gentle shaking (Figure S1, Supporting Information). The scanning electron microscope (SEM) images in Figure 2B show the intricate morphologies of both HAMA and SBHA microspheres after freeze‐drying. The microfluidic microspheres exhibited excellent dispersion under light microscopy, indicating their pristine morphological characteristics (Figure 2C). The particle sizes were verified using a Brookhaven 90 Plus DLS instrument, which displayed a narrow and uniform size distribution (Figure 2D). The hydrogel microspheres possessed an average dimension of ≈58.04 ± 29.46 µm. The spherical morphology of these microspheres enabled smooth rolling and also aided in ball‐bearing lubrication. A phase‐analysis light‐scattering technique was used to obtain the curve shown in Figure 2E. From this curve the zeta potential of microspheres was calculated to be −10.13 ± 1.11 mV, which enhanced their ability to carry positively charged drugs. To confirm the successful preparation of Met@SBHA, we measured the zeta potential of Met@SBHA, which was found to be −3.25 ± 1.16 mV. Metformin, a positively charged drug, was adsorbed onto the negatively charged microspheres through electrostatic attraction. Therefore, metformin was successfully loaded into the microspheres and prepared as Met@SBHA. The presence of the characteristic S 2s (169.8 eV) peak in the X‐ray photoelectron spectroscopy (XPS) analysis indicated effective surface modification of the copolymers on the hydrogel microspheres. This observation is further corroborated by the narrow elemental spectrum observed in the high‐resolution XPS analysis, as illustrated in Figure 2F. Moreover, the transformation ratio of the N 1s (402.0 eV) peak also provided evidence for the presence of coatings on the surface of the microspheres. Similarly, the microsphere composition was analyzed based on chemical bonding using Fourier Transform Infrared Spectroscopy (FTIR), which showed a distinct peak at 1640 cm^−1^ corresponding to a double bond in the microspheres, reflecting the interaction between hyaluronic acid and methacrylic anhydride. In addition, enhanced intensity peaks associated with sulfonate (SO_3_^−^) were detected at 600 cm^−1^ in SBHA compared to HAMA (Figure 2G). In summary, the aforementioned material characterizations provide strong evidence for the successful preparation of SBHA microspheres.

To investigate the lubricating characteristics of the SBHA microspheres, tribological tests were performed in contact‐pressurized mode, as shown in Figure 2H. Coefficient of friction (COF)–time plots and COF histograms are shown in Figure 2I,J, respectively. The COF of the hydrogel microspheres with and without the zwitterionic group SB‐NH2 was notably lower than that of PBS. Moreover, the COF of the drug‐loaded microspheres (Met@SBHA) showed no significant variance from the SBHA microspheres. Upon analyzing the resulting graph, it was observed that the coefficient of friction (COF) for the drug‐loaded microspheres was 0.035, showing no significant alteration when compared to the COF of SBHA microspheres at 0.034. This suggests that the friction characteristics of the microspheres remained unaffected following the drug loading process. Friction coefficients measured for whole joints in vivo have been documented to vary between about 0.001 and 0.03.^[^ ^25^ ^]^ The microspheres exhibited an enhanced load‐bearing capacity and lubrication efficiency owing to their significant elastic and rolling characteristics. Moreover, the lubricating capabilities of the SBHA microspheres surpassed those of the HAMA microspheres, thereby synergistically reducing the COF through hydration lubrication provided by the zwitterionic phosphocholine groups in the copolymers. Overall, the tribological tests confirmed that the application of SBHA microspheres effectively enhanced lubrication. Concurrently, the microspheres were loaded with the drug without compromising their intrinsic frictional properties.

The mechanical stability of microspheres under the cyclic loading of joint motion determines whether they can maintain a good apparent morphology and sustained drug release.^[^ ^26^, ^27^ ^]^ To investigate the mechanical properties of the synthesized microspheres, we investigated the surface morphology and surface roughness, DMT modulus and force profiles of the microspheres using atomic force microscopy AFM. It was evident that a decrease in the roughness of SBHA resulted in a smoother surface, with both the 2D and 3D profiles displaying remarkable uniformity (Figure S2, Supporting Information). This indicated that the smooth and uniform morphology of SBHA can more effectively facilitate the seamless movement of the joint space and significantly diminish friction. To evaluate the deformation of the microspheres under joint force, similarly, the force‐displacement curves plotted proved this feature. When the probe contacted the sample to generate a repulsive force, the distance dropped by the probe was linear with respect to the force, and using this law, we calculated that the Young's modulus on the surface of HAMA and SBHA microspheres were ≈7.09 kPa and 9.59 kPa respectively (Figure 2K, Figure S2 and Table S1, Supporting Information). Young's modulus is an indicator of a material's rigidity, describing its elastic responses to load‐deflection scenarios, and is gauged through tension, flexure, or compression. The greater the Young's modulus, the superior the material's stiffness. These results suggest that our fabricated microspheres exhibit satisfactory mechanical stability.

The ability to stabilize drug release and degradation properties of biomaterials for intra‐articular injection are important for the treatment of OA. Hydrogel microspheres, acting as proficient carriers, prevent the rapid depletion of medication and achieve sustained and localized release. Metformin (Met), a member of the biguanide class of hypoglycemic drugs with a positive zeta potential, possesses two interconnected guanidine rings. To confirm the successful preparation of Met@SBHA, we measured the zeta potential of the microspheres and Met@SBHA. Our results demonstrated the zeta potential of microspheres was −10.13 ± 1.11 mV, whereas that of Met@SBHA was determined to be −3.25 ± 1.16 mV (Figure 2E and Figure S3, Supporting Information). Additionally, the UV spectrum full wavelength scan and the standard curve of Met in PBS were illustrated in Figure S4, Supporting Information. The drug loading efficiency of Met@SBHA was calculated as 76.64% based on the standard curve and UV absorbance of the drug. These results validated the successful preparation of Met@SBHA. The drug release profile was illustrated in Figure 2L. The slope of the curve was notably large during the first 2 weeks, indicating a relatively rapid phase of drug release. Subsequently, the curve tended to flatten, and drug release gradually decreased, entering a relatively stable release phase. This result suggests that Met@SBHA exhibited sustained and stable drug release behavior, which facilitated long‐term drug delivery to the lesion after injection. As shown in Figure 2M, under the influence of collagenase in a simulated in vivo environment, the residual time of Met@SBHA was as long as 28 d, indicating that the slower biodegradation rate of Met@SBHA can prolong the retention time in vivo, contributing to sustained lubrication and therapeutic effects.

### 2.2 Cytotoxicity and Cytocompatibility of Met@SBHA Were Assessed In Vitro

**Figure 3:**
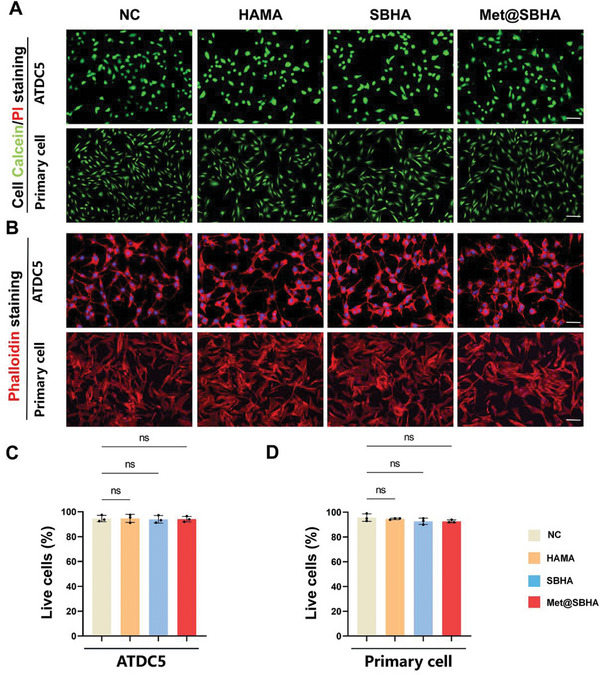
In vitro assessment of the cytotoxicity and cytocompatibility of Met@SBHA. A) The live/dead staining of ATDC5 cell line and primary chondrocytes after 24 h co‐culture with different hydrogel microspheres (HAMA, SBHA and Met@SBHA). Scale bars: 500 µm. B) Cytoskeleton staining images of cells co‐cultured with different hydrogel microspheres. Scale bars: 500 µm. C, D) Quantitative analysis of the live/dead staining. Number of repetitions of all experiments n ≥ 3. Data are presented as the mean ± SD. (*P < 0.05; **P < 0.01; ***P < 0.001 and ****P <0.0001).

The Met dosage screening of Met@SBHA was performed prior to the initiation of the respective experiments, determining 1 mM as the optimal metformin dosage encapsulated within the microspheres (Figure S5, Supporting Information). To ascertain the feasibility of employing Met@SBHA in OA therapy, it was imperative to examine the cytotoxicity and cytocompatibility of Met@SBHA in chondrocytes in vitro. As depicted by live/dead staining (**Figure** **3**A) and quantitative analysis (Figure 3C,D), the viability of the ATDC5 cell line and primary cartilage cells in all hydrogel microsphere groups remained above 90% after a 24‐h incubation, indicating the favorable cytocompatibility of the hydrogel microspheres. The morphology of cartilage is an essential factor in the formation of a smooth surface within the joint. No discernible variations in cell morphology were observed upon incubation with distinct hydrogel microspheres compared to the control group (Figure 3B). Significantly, the hemolysis ratio values exhibited by all hydrogel microspheres were below 2%, a significantly lower threshold than the critical safe hemolysis ratio (5%) established for biomaterials, as defined in the ASTM standard (F756‐2008) (Figure S6A,B, Supporting Information). Furthermore, in the positive group, erythrocyte debris were distinctly discernible because of their complete rupture, whereas in the other groups, erythrocytes exhibited a biconcave discoidal morphology (Figure S6C, Supporting Information). These results show the nontoxic and biocompatible properties of Met@SBHA, suggesting its potential as a viable injectable material for OA treatment.

### 2.3 Met@SBHA Attenuates Chondrocyte Senescence In Vitro

**Figure 4:**
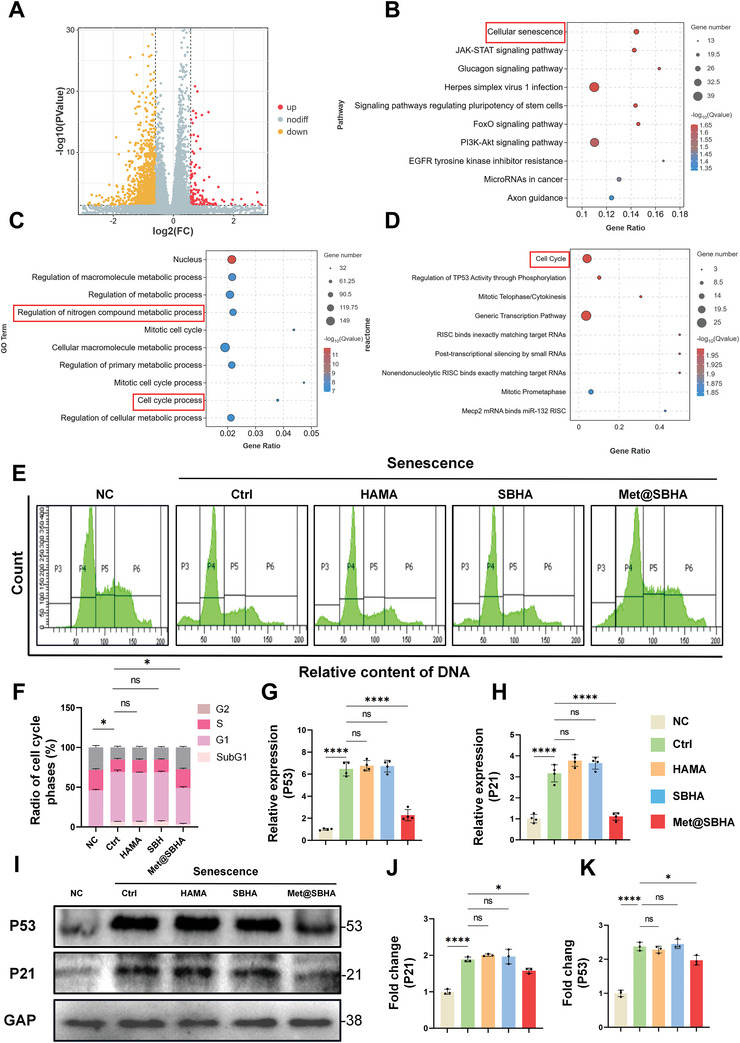
Met@SBHA alleviates chondrocyte senescence in vitro. A) Volcano plot of the distribution of differentially expressed genes (DEGs). B) Reactome enrichment analysis of DEGs. C) GO enrichment analysis of DEGs. D) KEGG enrichment analysis of DEGs. E) Distribution plot of the DNA content of the cell cycle analyzed by flow cytometry (FCM). F) Statistical plots of the quantitative analysis of the cell cycle analyzed by flow cytometry (FCM). G,H) RT‐PCR results showing the relative mRNA expression levels of P53 and P21. I–K) Representative immunoblot analysis and quantification of P53 and P21 protein expression in treated chondrocytes. The subgroups are as follows: NC group = normal cells; Ctrl group = senescent cells without any treatment; HAMA group = senescence cells treated with hyaluronic acid methacrylate microspheres; SBHA group = senescence cells treated with sulfobetaine (SB)‐lubricated HAMA microspheres; Met@SBHA group = senescence cells treated with metformin‐encapsulated SBHA microspheres. Number of repetitions of all experiments n ≥ 3. Data are presented as the mean ± SD. (*P < 0.05; **P < 0.01; ***P < 0.001 and ****P <0.0001).

Recent studies have highlighted the notable effectiveness of Met in alleviating primary manifestations of biological senescence,^[^ ^28^ ^]^ establishing it as an exceptionally compelling candidate in the field of antiaging. However, the ability of Met to alleviate chondrocyte senescence remains unclear. To gain insight into the impact of Met on chondrocyte senescence, we conducted mRNA sequencing analysis of primary senescent chondrocytes and primary senescent chondrocytes subjected to Met@SBHA treatment. In comparison to the senescent group, a comprehensive count of 1309 differentially expressed genes (DEGs) was detected in the Met@SBHA group, with 144 genes displaying upregulation and 1165 genes showing downregulation (P < 0.05, |log2 [fold change] | > 1.5) (**Figure** **4**A). Kyoto Encyclopedia of Genes and Genomes (KEGG) pathway analysis revealed that DEGs in both groups were significantly associated with functional annotations pertaining to cellular senescence, signaling pathways governing the pluripotency of stem cells, and Foxo signaling pathways (Figure 4B). These pathways play crucial roles in cartilage repair and regulation of senescence. Gene Ontology (GO) and reactome pathway enrichment analyses indicated a robust association between DEGs and the regulation of cellular metabolism, nitrogen compound metabolism, and cell cycle progression in both groups (Figure 4C,D). As shown in Supporting Information of Figure S7 (Supporting Information), the corresponding cellular senescence and cell cycle were upregulated in the GSEA enrichment analysis. Therefore, cell cycle functions were analyzed. Cell cycle distribution was assessed using flow cytometry (FCM), as shown in Figure 4E,F. The results revealed a notable increase in the proportion of cells in the S phase when the senescent chondrocytes were treated with Met@SBHA (ranging 12.3–24.5%). The number of cells in the G1 phase was as follows: NC (48.5%) Ctrl, (62.5%); HAMA (63.2%); SBHA (62.7%); and Met@SBHA (42.6%), respectively. These findings suggest that treatment with Met@SBHA facilitates the transition of senescent chondrocytes from the G1 phase to the S phase, thereby profoundly stimulating cellular proliferation.

**Figure 5:**
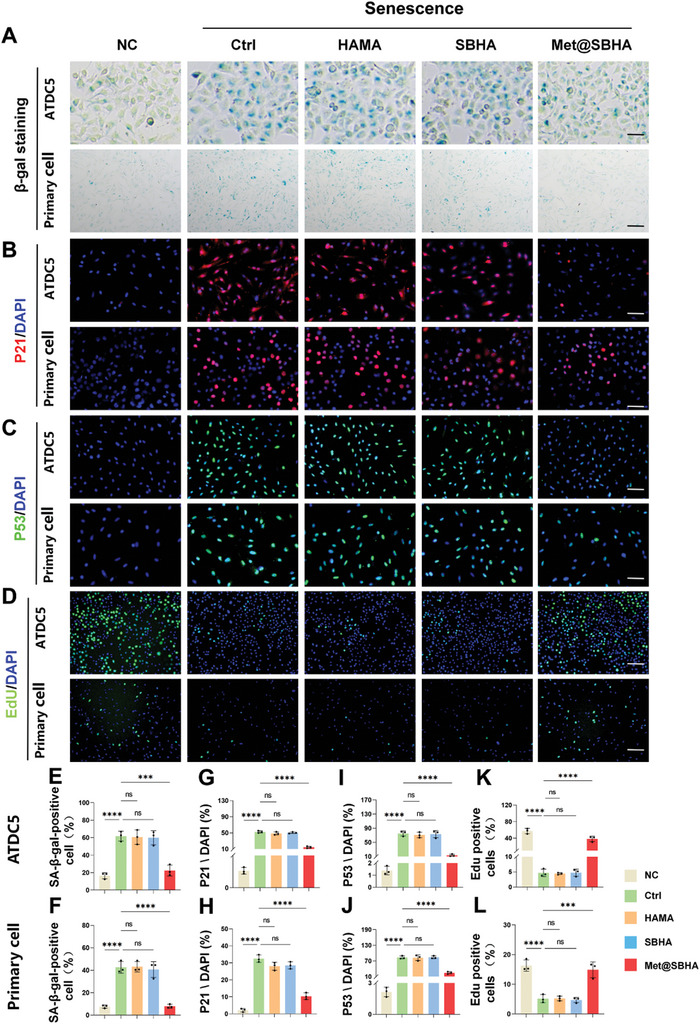
Met@SBHA alleviates chondrocyte senescence in vitro. A) SA‐β galactosidase staining of ATDC5 cell line (Scale bars: 100 µm) and primary chondrocytes (Scale bars: 500 µm). B,C) Immunofluorescence staining of P21 and P53 in ATDC5 cell line and primary chondrocytes. Scale bars: 500 µm. D) Edu staining of ATDC5 cell line and primary chondrocytes. Scale bars: 500 µm. E–L) Statistical quantification of the SA‐β‐gal‐positive chondrocytes, P21 and P53 positive expression cells, and EdU positive cells in ATDC5 chondrocyte cell line and primary chondrocytes separately. Number of repetitions of all experiments n ≥ 3. Data are presented as the mean ± SD. (*P < 0.05; **P < 0.01; ***P < 0.001 and ****P <0.0001).

The expression levels of pivotal senescence‐related genes, such as P53 and P21, were further assessed (Figure 4G,H). As illustrated by the western blot findings (Figure 4I–K), a decrease in the expression of P53 and P21 was observed in the Met@SBHA group compared to the Ctrl, HAMA, and SBHA groups. Senescence‐related staining (**Figure** **5**A–C) and quantitative analysis (Figure 5E–J) elucidated parallel findings, showcasing a notable decline in β‐gal, P21, and P53, while exhibiting an augmentation in EdU staining (Figure 5D,K,L) within the Met@SBHA group, when compared with the Ctrl, HAMA, and SBHA groups. Nevertheless, no discernible discrepancy was observed when compared with the NC group. Cellular senescence is often accompanied by the production of senescence‐associated secretory phenotype (SASP),^[^ ^29^ ^]^ and the expression of pro‐inflammatory cytokines IL‐1β and IL‐6 was significantly decreased in the Met@SBHA group compared with the senescent chondrocyte group (Figure S8, Supporting Information). These results indicate that Met@SBHA possesses a significant ability to counteract chondrocyte senescence.

### 2.4 Met@SBHA Alleviates Chondrocyte Senescence Via iNOS/ONOO‐/P53 Axis

**Figure 6:**
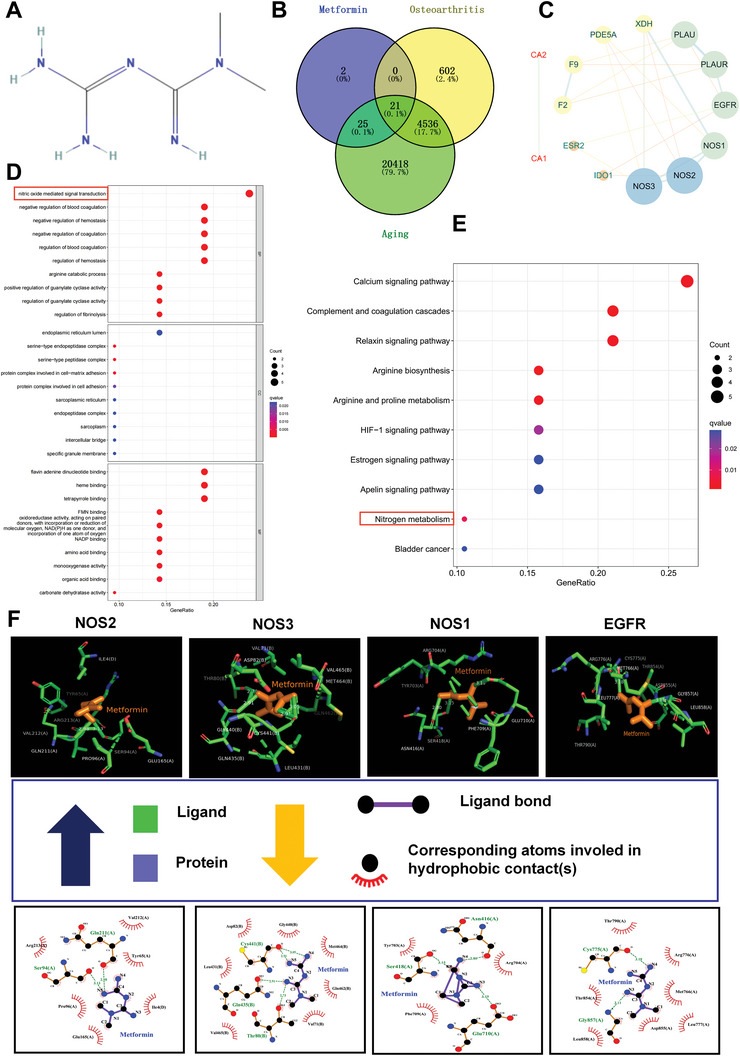
Network pharmacological analyses and molecular docking reveal NOS2 (iNOS) as target gene for the regulatory effects of Met over senescence. A) 2D molecular structure of Met. B) Venn diagram of the intersection of osteoarthritis, senescence, and Met‐associated genes. C) Protein‐Protein Interaction (PPI) networks reveals 12 core gene targets. D) GO analysis of core gene targets. E) KEGG analysis of core gene targets. F) The binding site and site‐preceding interactions of Met and pre‐4 hub proteins (NOS2, NOS1, NOS3, and EGFR).

To elucidate the mechanisms underlying the therapeutic effects of Met on chondrocyte senescence, we conducted a comprehensive network pharmacological analysis. The exquisite two‐dimensional molecular structure of Met was meticulously retrieved from the esteemed PubChem database (**Figure** **6**A), and a total of 48 pharmacological targets were astutely predicted through the employment of the SwissTargetPrediction databases. To ensure the veracity of our findings, we used the UniProt database to eliminate superfluous genes. Furthermore, the Genecard database revealed 5159 genes with significant relevance to OA, along with 25000 genes significantly associated with the aging process. Subsequently, 21 overlapping genes associated with MET in relation to both OA and aging were identified (Figure 6B). Using the MCODE algorithm, we extracted the most noteworthy modules from the protein‐protein interaction (PPI) network. Using cytoHubba, we identified 12 core gene targets: NOS2, NOS3, NOS1, EGFR, PLAUR, PLAU, XDH, PDE5A, F9, F2, ESR2, and IDO1, as illustrated in Figure 6C). Gene analysis findings implied that Met potentially influenced a range of cellular mechanisms, including the transduction of signals mediated by nitric oxide and the regulation of coagulation and hemostasis (Figure 6D). The investigation revealed ten KEGG pathways that exhibited significant enrichment, with a P‐adjusted value below 0.05. These pathways included the calcium signaling pathway, HIF‐1 signaling pathway, arginine biosynthesis, estrogen signaling pathway, and nitrogen metabolism (Figure 6E). Molecular docking analysis was conducted in the current study to investigate the potential binding interactions between Met and key targets. The findings indicated that NOS2, NOS3, NOS1, and EGFR were the top four targets with the highest binding affinities for Met (Figure 6F; Figure S9, Supporting Information). Docking scores ranging −6.24 – −4.55 were obtained for 12 compounds (Table S2, Supporting Information). Combined with the results of the mRNA sequencing analysis, it was demonstrated that Met can govern the intricate process of nitrogen metabolism. Therefore, NOS2 (iNOS) was selected as the target gene through which Met exerts its regulatory influence on senescence.

**Figure 7:**
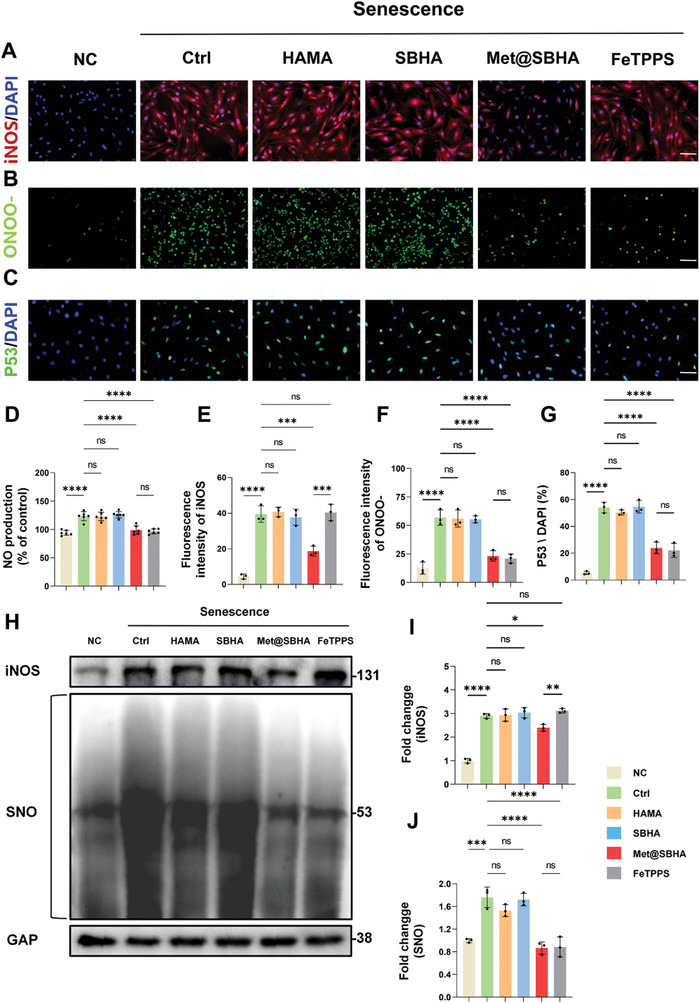
Met@SBHA attenuates chondrocyte senescence by affecting the iNOS/ONOO‐/P53 pathway. A) Immunofluorescent staining of iNOS in primary chondrocytes (Scale bars: 500 µm). B) Expression of fluorescent probe‐labelled peroxynitrite (ONOO‐) in chondrocytes. (Scale bars: 500 µm). C) Immunofluorescent staining of P53 in primary chondrocytes (Scale bars: 500 µm). D) Quantitative analysis of NO production in the supernatants of each cell group. E) Quantitative analysis of iNOS fluorescence intensity. F) Quantitative analysis of the fluorescence intensity of ONOO‐. G) Quantitative analysis of the percentage of P53 positive expression cells. H–J) Representative immunoblot analysis and quantification of iNOS and *S*‐nitrosylated proteins expression. NC = normal cells; Ctrl = senescent cells without any treatment; HAMA = senescence cells treated with HAMA; SBHA = senescence cells treated with SBHA; Met@SBHA = senescence cells treated with Met@SBHA; FeTPPS = senescence cells treated with FeTPPS. Number of repetitions of all experiments n ≥ 3. Data are presented as the mean ± SD. (*P < 0.05; **P < 0.01; ***P < 0.001 and ****P <0.0001).

To ascertain the effect of Met on iNOS expression in senescent chondrocytes, we conducted additional assessments of iNOS expression after treatment with Met@SBHA. As depicted in **Figure** **7**A,E, a notable decrease in the relative mRNA expression levels of iNOS was observed following Met@SBHA treatment in contrast to the control, HAMA, and SBHA groups. However, no substantial disparities were observed compared to the NC group. Nitric oxide (NO) serves as a pivotal intercellular messenger synthesized from l‐arginine through the catalytic action of iNOS. Nitric oxide (NO) is widely regarded as a critical signaling molecule that controls a myriad of essential cellular processes while also acting as a potent mediator of cellular harm.^[^ ^30^ ^]^ We investigated whether NO generation was involved in Met@SBHA treatment. Notably, the Met@SBHA group displayed a notable suppression of NO generation under senescent conditions, closely resembling the observations made in the NC group (Figure 7D). Notably, NO can combine with superoxide (O2‐) to form the more potent oxidant, peroxynitrite (ONOO‐). Intracellular levels of ONOO‐ were assessed using a fluorescent probe ONOO‐ test kit. The results revealed that treatment with Met@SBHA decreased intracellular ONOO‐ generation, exhibiting a pattern similar to that observed in the NC group. Moreover, there was no significant difference when compared to the group treated with ONOO‐ remover (FeTPPS group) (Figure 7B,F). These findings suggest that Met@SBHA can mitigate the expression of iNOS, thereby further inhibiting the generation of NO and production of intracellular ONOO‐. Previous studies have demonstrated that ONOO‐ plays a pivotal role in regulating the expression of numerous genes, and its influence partly mediates *S*‐nitrosylation.^[^ ^31^ ^]^ *S*‐nitrosylation of a specific domain within a transcription factor can modulate its activation, protein stability, and subcellular localization, as exemplified by the case of P53.^[^ ^32^ ^]^ P53 is a critical transcription factor that governs the expression of various proteins involved in cell cycle regulation and senescence.^[^ ^33^ ^]^ As shown in the immunofluorescence images in Figure 7C,G, the reduction in ONOO‐ was accompanied by a significant reduction in P53 protein expression in chondrocytes. To validate the effect of Met@SBHA on the induction of *S*‐nitrosylation within the cells, we assessed the generation of *S*‐nitrosylated proteins in response to Met@SBHA. Specific detection of *S*‐nitrosylated proteins was accomplished using the Pierce *S*‐nitrosylation western blot assay. The results revealed a significant increase in *S*‐nitrosylated proteins in senescent cells compared to the control group (Figure 7H–J). Moreover, Met@SBHA exhibited the ability mitigated the expression of *S*‐nitrosylated proteins in senescent cells. Collectively, these findings indicate the potential of Met@SBHA to alleviate chondrocyte senescence by inhibiting iNOS, thereby exerting a consequential effect on the iNOS/ONOO‐/P53 pathway.

iNOS serves as a proinflammatory factor,^[^ ^34^ ^]^ and prior research has revealed that Piezo1 channels confer high strain mechanosensitivity to articular cartilage, and activation of mechanosignaling pathways mediated by Piezo1 proteins sensing and responding to mechanical stimuli regulates chondrocyte damage and accelerates OA progression.^[^ ^35^, ^36^ ^]^ Therefore, we delved deeper into the alteration of mechanotransduction pathways in senescent chondrocytes. Our findings illustrated an upregulation of Piezo1 expression and its associated influx of Ca^2+^ in senescent chondrocytes, which was mitigated by Met@SBHA (Figure S10, Supporting Information).

### 2.5 Met@SBHA Orchestrates the Metabolic Equilibrium of Chondrocytes In Vitro

**Figure 8:**
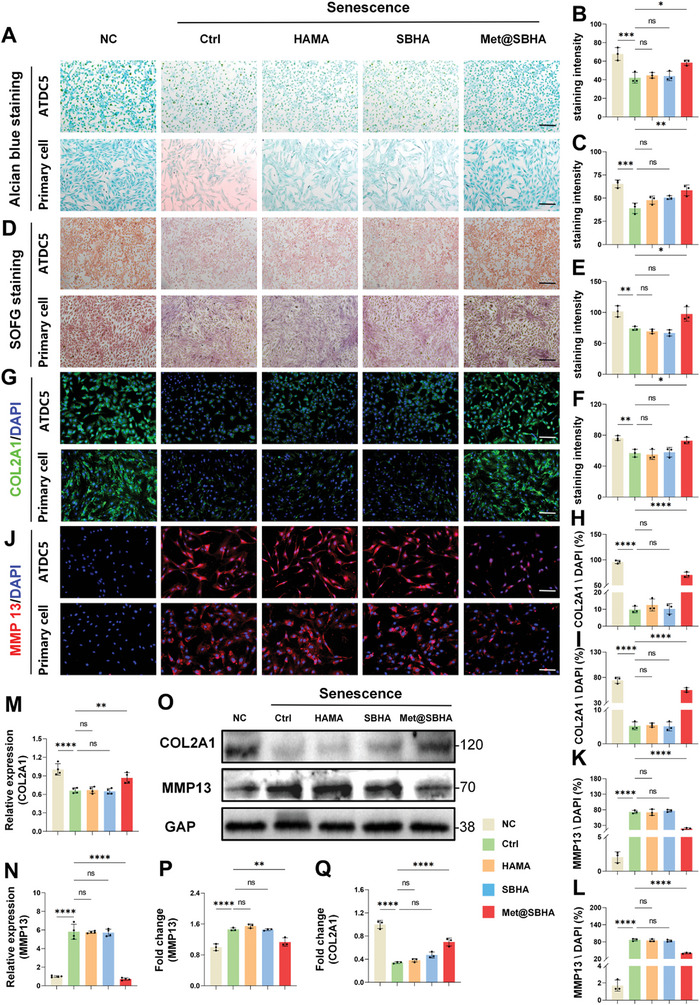
Met@SBHA coordinates the metabolic homeostasis of chondrocytes in vitro. A–C) Alcian blue staining and its quantitative analysis of ATDC5 cell line and primary chondrocytes (Scale bars: 500 µm). D–F) Safranin O‐Fast Green staining and its quantitative analysis of ATDC5 cell line and primary chondrocytes (Scale bars: 500 µm). G–I) Immunofluorescence staining and its quantitative analysis of COL2A1 in ATDC5 cell line and primary chondrocytes (Scale bars: 500 µm). J–L) Immunofluorescence staining and its quantitative analysis of MMP13 in ATDC5 cell line and primary chondrocytes (Scale bars: 500 µm). M,N) Relative mRNA expression levels of COL2A1 and MMP13. P,Q) Representative immunoblot analysis and quantification of COL2A1 and MMP13 protein expression in NC group, Ctrl group, HAMA‐treated chondrocytes group, SBHA‐treated chondrocytes group, and Met@SBHA‐treated chondrocytes group. Number of repetitions of all experiments n ≥ 3. Data are presented as the mean ± SD. (*P < 0.05; **P < 0.01; ***P < 0.001 and ****P <0.0001).

The degeneration of cartilage involves the unfortunate demise or depletion of chondrocytes, as well as the loss of proteoglycans.^[^ ^37^ ^]^ Various hydrogel microsphere treatments were used to assess cartilage degeneration under senescent conditions. Alcian blue and Safranin O‐fast Green staining were performed to examine the secretion of cartilage matrix under various hydrogel microsphere treatments in the senescent milieu. As illustrated in **Figure** **8**A, the Met@SBHA group exhibited round blue stained masses, which displayed a slight fading of color, but remained comparable to those observed in the NC group. In contrast, only scattered stained particles were detected in the control, HAMA, and SBHA groups. Based on the quantification of Alcian blue staining (Figure 8B,C), the Met@SBHA group exhibited a significantly more profound relative color intensity of cell matrix staining than the control, HAMA, and SBHA groups. However, there was no significant difference compared with the NC group. Similarly, Safranin O‐fast green staining (Figure 8D–F) revealed a heightened intensity of Safranin O staining in the Met@SBHA group, indicating an elevated concentration of proteoglycan within the cartilage cells. This observation serves as an indicator of chondrogenesis. Immunofluorescent staining was performed to assess the metabolic balance within the cartilage. Notably, the Met@SBHA group exhibited a significant up‐regulation of COL2A1 (collagen type II alpha 1 chain) (Figure 8G–I) and a significant reduction in MMP13 levels under senescent conditions (Figure 8J–L), closely resembling those observed in the NC group. At the gene expression level, COL2A1 and MMP13 were validated in terms of both mRNA expression and quantitative analysis of immunoblotted proteins (Figure 8M–Q). Aggrecan, a major structural component of the extrachondral matrix, and SOX9, which regulates cartilage development, showed a significant increase in mRNA expression in Met@SBHA‐treated chondrocytes (Figure S11, Supporting Information). Consequently, Met@SBHA effectively maintained the metabolic balance in senescent chondrocytes, thereby mitigating cartilage degradation.

### 2.6 In Vivo Biosafety of Met@SBHA in Aged Mice

**Figure 9:**
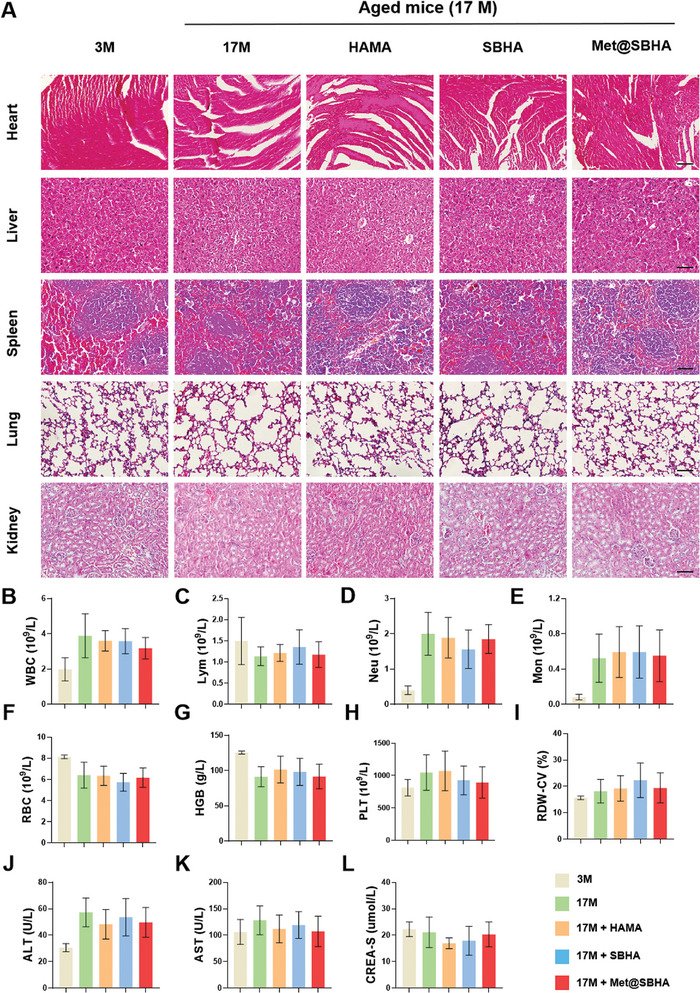
In vivo biocompatibility and biosafety of Met@SBHA. A) Staining of HE sections of mouse heart, liver, kidney and lung in mice at the age of 3 months (3 M group), mice at the age of 17 months (17 M group), 17‐month mice treated with HAMA (HAMA group), 17‐month mice treated with SBHA (SBHA group), 17‐month mice treated with Met@SBHA (Met@SBHA group). Scale bar 100 µm. B–L) Serum levels of biomarkers reflecting liver and kidney function and hematological parameters in mice. Number of repetitions of all experiments n = 6. Data are presented as the mean ± SD. (*P < 0.05; **P < 0.01; ***P < 0.001 and ****P <0.0001).

The in vivo imaging systems (IVIS) were initially employed to evaluate the retention time of fluorescent FITC‐labeled microspheres. Our findings reveal a gradual decline in the FITC fluorescence signal of Met@SBHA, which remained detectable even after a duration of 28 days (Figure S12, Supporting Information), confirming the admirable biodegradation efficacy and stable retention of Met@SBHA in vivo. Subsequently, we assessed the in vivo biocompatibility and safety of Met@SBHA using H&E staining, hematological examinations, and biochemical analyses. Histological examination of the organ sections using H&E staining demonstrated the absence of any discernible indications of toxicity in all experimental groups, thereby affirming the commendable safety profile of Met@SBHA (**Figure** **9**A). Hematological parameters, including white blood cell (WBC), neutrophil (Neu), lymphocyte (Lym), monocyte (Mon), red blood cell (RBC), hemoglobin (HGB), platelet (PLT), and red blood cell distribution width (RDW) counts were within the normal range in all groups (Figure 9B–I). The liver and kidney functions, as indicated by the levels of alanine aminotransferase (ALT), aspartate aminotransferase (AST), and blood creatinine (CR), remained within the normal range (Figure 9J–L), further confirming the excellent in vivo biocompatibility and safety of Met@SBHA.

### 2.7 Met@SBHA Ameliorates the Process of Cartilage Senescence in Aged Mice

**Figure 10:**
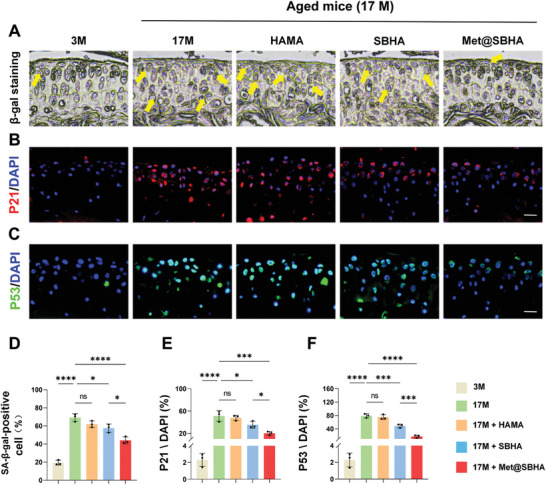
Met@SBHA attenuates the aging process of cartilage in aged mice. A) SA‐β galactosidase staining of the cartilage layer in 3 M group, 17 M group, 17M+HAMA group, 17M+SBHA group, and 17M+Met@SBHA group. Scale bar 100 µm. B) Immunofluorescence staining to detect the expression of P21 in the cartilage layer in 3 M, 17 M, HAMA group, SBHA group, and Met@SBHA group. Scale bar 100 µm. C) Immunofluorescence staining of P53 in cartilage layers. Scale bar 100 µm. D–F) Quantitative analysis of the percentage of SA‐β‐gal‐positive chondrocytes, P21‐positive expressing cells and P53‐positive expressing cells in cartilage layer. Number of repetitions of all experiments n ≥ 3. Data are presented as the mean ± SD. (*P < 0.05; **P < 0.01; ***P < 0.001 and ****P <0.0001).

We investigated the potential of the intra‐articular administration of Met@SBHA to the knee joints of aged mice to alleviate cartilage senescence. Mice were divided into five groups: 3 M, 17 M, 17M+HAMA, 17M+SBHA, and 17M+Met@SBHA groups. The hydrogel microspheres were administered to mice at the age of 15 months, and all mice were harvested at 17 months of age. The senescence of cartilage was assessed through β‐gal staining and immunofluorescence staining of P53 and P21. The β‐gal staining revealed a reduction in the number of senescent cells in the SBHA and Met@SBHA groups, compared to the 17 M and HAMA groups (**Figure** **10**A,D). However, there were no significant differences compared to the 3 M group. In addition, the SBHA and Met@SBHA groups exhibited a notable decrease in the levels of P21 (Figure 10B,E) and P53 (Figure 10C,F) compared to the 17 M and HAMA groups. Nonetheless, no evident disparities were noted when compared to the 3 M group. Notably, the Met@SBHA group achieved superior outcomes compared with the SBHA group, demonstrating the advantages of enhancing lubrication and combating aging. Overall, these findings suggested that Met@SBHA effectively alleviated cartilage senescence in aged mice.

### 2.8 Met@SBHA Attenuates the Pathogenesis of Age‐Associated OA

**Figure 11:**
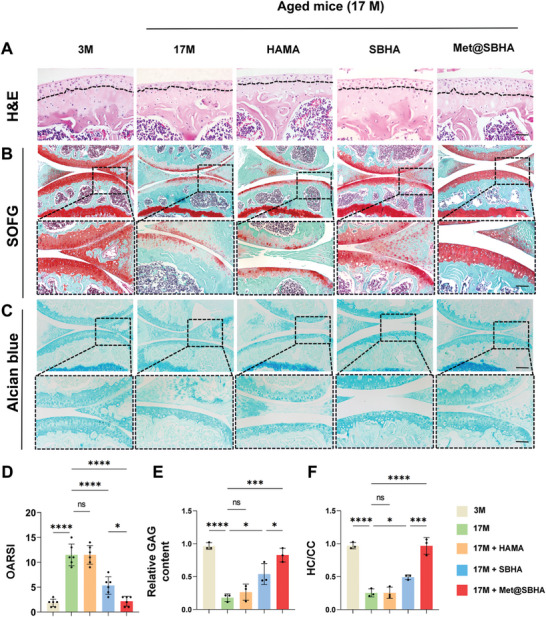
Met@SBHA alleviates effectively cartilage degeneration in age‐related osteoarthritis A) Representative images of HE staining in 3 M group, 17 M group, 17M+HAMA group, 17M+SBHA group, and 17M+Met@SBHA group. Scale bar: 100 µm. B) Safranin O‐fast green staining (upper panel) with enlarged area in the upper box of the subchondral bone area (middle panel). Scale bar: top 100 µm; bottom 50 µm. C) Alcian blue staining in each group. Scale bar: top 100 µm; bottom 50 µm. D) OARSI score of each group; n = 6 E) Quantitative analysis of relative glycosaminoglycan (GAG) content. F) The ratio of hyaline cartilage (HC) and calcified cartilage (CC) of articular cartilage. Number of repetitions of all experiments n ≥ 3. Data are presented as the mean ± SD. (*P < 0.05; **P < 0.01; ***P < 0.001 and ****P <0.0001).

**Figure 12:**
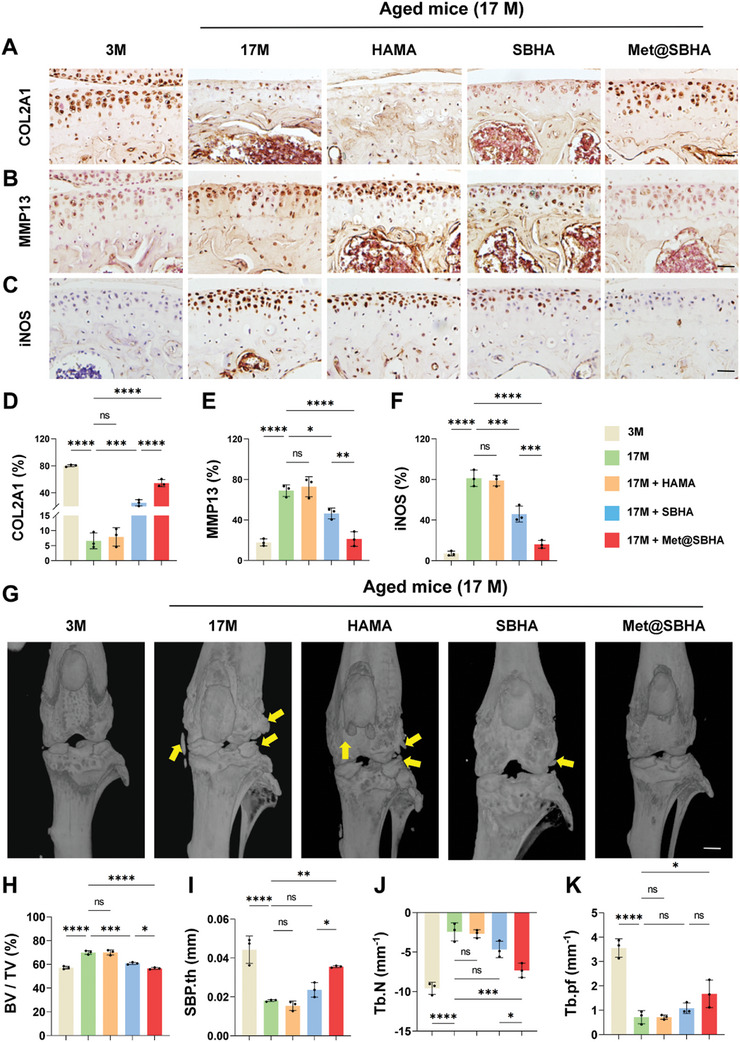
Met@SBHA attenuates the pathogenesis of age‐associated OA. A, B) Immunohistochemical staining for MMP13 and COL2A1 for assessing the metabolic balance of cartilage. Scale bar 100 µm. C) Immunohistochemical staining of the screened target protein iNOS. Scale bar 100 µm. D, F) Quantitative analysis of immunohistochemical staining of MMP13, COL2A1, and iNOS. G) Micro‐CT images of medial subchondral bone. Scale bar: 500 µm. H–K) Quantitative analysis of BV/TV (bone tissue relative to the total tissue volume), SBP.th (Subchondral Bone Plate thickness), Tb.pf (trabecular pattern factor) and Tb.Th (trabecular thickness) in the subchondral bone of 3 M group, 17 M group, 17M+HAMA group, 17M+SBHA group, and 17M+Met@SBHA group. Number of repetitions of all experiments n ≥ 3. Data are presented as the mean ± SD. (*P < 0.05; **P < 0.01; ***P < 0.001 and ****P <0.0001).

To further ascertain the potential of Met@SBHA in retarding the progression of OA, histological staining assays were performed on cartilage tissues, encompassing a range of techniques such as hematoxylin‐eosin (H&E) staining, Safranin O‐fast green staining, Masson staining, and Alcian blue staining. As illustrated in **Figure** **11**A–C, the 17 M group exhibited characteristic cartilage features of OA, including surface discontinuity and erosion fissures. The group treated with HAMA showed no notable differences compared to the aged group. However, both SBHA and Met@SBHA groups demonstrated significant enhancements in morphological changes, matrix staining, and tidemark integrity. Met@SBHA was regarded as the most efficacious treatment option, exhibiting the most favorable OARSI score compared with the other treatments (Figure 11D). In addition, the depth of the cartilage lesions was significantly diminished in the SBHA and Met@SBHA groups in comparison to the 17 M and HAMA groups, as evidenced by the HC/CC ratio and relative glycosaminoglycan (GAG) content of each group (Figure 11E). Notably, the Met@SBHA group displayed the best results in terms of the HC/CC ratio among the various treatment groups, exhibiting a significant 80% increase relative to that of the 17 M group (Figure 11F). Masson's staining showed that Met@SBHA significantly alleviated fibrosis in the cartilage (Figure S13, Supporting Information). Immunohistochemical staining was used to evaluate metabolic equilibrium within the cartilage. The Met@SBHA group demonstrated the most favorable outcome in terms of the upregulation of COL2A1 (**Figure** **12**A,D) and a substantial decrease in MMP13 levels (Figure 12B,E) compared to the other groups. The results obtained from the iNOS staining were consistent (Figure 12C,F). Overall, these findings indicated that Met@SBHA effectively mitigated cartilage deterioration in elderly mice.

An ever‐growing body of evidence indicates that the progressive deterioration of the subchondral bone plays a pivotal role in OA development, acting as a catalyst for subsequent degeneration of the overlying cartilage.^[^ ^38^ ^]^ We subsequently explored the modification of the subchondral bone in elderly mice subjected to various treatments. As evidenced by the microcomputed tomography (micro‐CT) scans, the knee joints in the groups treated with SBHA and Met@SBHA displayed minimal osteophytes, comparable to those in the 3 M group. In contrast, osteophytes were prominently observed in the 17‐month and HAMA groups (Figure 12G–K). Moreover, the thickness of the synovial lining layer and the total synovitis scores in the SBHA and Met@SBHA groups were similar to those in the 3 M group, but significantly lower than those in the 17 M and HAMA groups (Figure S14, Supporting Information). Notably, the Met@SBHA group exhibited superior outcomes in terms of mitigating cartilage degeneration, subchondral bone deterioration, and synovial alteration compared with the SBHA group, implying that Met@SBHA effectively impedes the advancement of OA by virtue of its dual advantages of enhanced lubrication and anti‐aging properties. Taken together, our exploration of the in vivo application of Met@SBHA revealed its potential as a promising therapeutic intervention for age‐related OA.

## 3 Discussion

In this study, we successfully developed an innovative microsphere by blending a zwitterionic polymer and HAMA to serve as an injectable substance for the effective management of age‐related OA. A growing body of literature presents compelling evidence for the notable effectiveness of zwitterionic surfaces in mitigating interfacial friction. Zwitterionic entities display an extraordinary affinity for water molecules, allowing the formation of a resilient hydration layer that resists dehydration and sustains elevated pressures.^[^ ^39^ ^]^ This fosters the establishment of enduring surface lubrication. Our study revealed that the integration of the zwitterionic group of SB reduced the COF of HAMA microspheres. This outcome can be ascribed to the formation of hydration shells surrounding the charged entities present in SBHA, specifically the positively charged quaternary ammonium group (NR_4_^+^) and the negatively charged sulfur trioxide group (SO_3_^−^). Because of their inherent dipoles, water molecules exhibit a pronounced affinity toward the charged groups on SBHA, thereby facilitating the creation of delicate H_2_O films on the hydrogel surface at the molecular level. This phenomenon leads to a notable decrease in the generation of shear stress between opposing surfaces and a consequent decline in the COF. Other researchers have also endeavored to conceive innovative approaches for enhancing the lubrication of hydrogel microspheres. For instance, CAP/FGF18‐hyEXO was encapsulated in HAMA hydrogel microspheres via microfluidics and photopolymerization to form an injectable microgel system (CAP/FGF18‐hyEXO@HMs). The system includes a regenerative hydration layer that provides continuous lubrication to cope with frictional wear.^[^ ^40^ ^]^ Additionally, some researchers fabricated GelMA@DMA‐MPC lubricated microspheres by dip‐coating DMA‐MPC, a self‐adhesive polymer synthesized through free‐radical co‐polymerization, onto the surface of GelMA photocrosslinked methacrylate gelatin hydrogel microspheres prepared via microfluidic technology.^[^ ^41^ ^]^ However, mere adjustments to the surface lubrication of microspheres may be insufficient for long‐lasting lubrication, due to the gradual biodegradation and wear of microspheres during joint motion. Nanospheres are considered as another crucial approach for the enhancement of lubrication,^[^ ^42^, ^43^ ^]^ with the primary concern being the rapid absorption feature during local administration. Interestingly, Dynamic Hydrogels, due to their ability to react to localized mechanical forces and biological cues with temporal and spatial accuracy, are attracting increasing attention in in both fundamental and translational biomedical research.^[^ ^44^, ^45^, ^46^ ^]^ However, with only a limited number of studies assessing dynamic hydrogels in vivo, there exists a pressing necessity to explore deeper into the complexity associated with the in vivo utilization of the dynamic hydrogels. In this study, we aimed to modify the amphiphilic ions SBs on HAMA to lubricate and modify the hydrogel as a whole. This modification helps alleviate joint wear caused by the reduction of synovial fluid in age‐related OA. SBHA can reduce surface friction by forming a hydrated surface, which produces long‐term lubrication effects, even during the gradual biodegradation of the hydrogel microspheres. Hence, we envision that the zwitterionic microsphere holds great promise as a formidable contender in augmenting the lubricity for the management of age‐related OA.

Metformin (Met), also referred to as *N*, *N*‐dimethylbiguanide, consists of two interconnected guanidine rings and is a biguanide hypoglycemic medication.^[^ ^47^ ^]^ Recent findings have revealed that Met exerts effects beyond its blood sugar‐lowering capabilities, such as mitigating the aging process through a multitude of mechanisms.^[^ ^48^ ^]^ These mechanisms involve the activation of AMPK and SIRT1, suppression of mTORC1, inhibition of NF‐κB and inflammatory pathways, modulation of gut microbiota, enhancement of metabolism, prevention of DNA damage, facilitation of DNA repair, and reduction of telomere shortening.^[^ ^48^, ^49^, ^50^ ^]^ One recent study has uncovered that Met possesses the ability to alleviate the symptoms of OA through its inhibition of the inflammatory response and reduction of apoptosis in chondrocytes.^[^ ^51^ ^]^ Additionally, another study has demonstrated that sustained delivery of Met in the joint cavity, supported by the use of a DNA supramolecular hydrogel, alleviated the development of traumatic OA.^[^ ^52^ ^]^ However, it should be noted that the OA model employed in these studies is a traumatic OA model, and the mice utilized are relatively young with an optimal state of cartilage homeostasis. Moreover, age‐related OA primarily arises from the degeneration and wear of joint cartilage, as well as a decline in joint lubricating fluid due to the natural process of aging. The mechanism by which Met provides cartilage protection in post‐injury OA models cannot be extrapolated to imply similar efficacy in age‐related OA. Consequently, the potential of Met to alleviate age‐related OA and the underlying mechanism of its treatment in this context remain unclear. In this study, we found that the administration of Met (Met@SBHA) mitigated the deterioration of cartilage by diminishing the senescence of chondrocytes compared to untreated aged mice. These findings provide substantial evidence supporting the notion that a reduction in the number of senescent chondrocytes plays a pivotal role in alleviating the progression of age‐associated OA and maintaining the delicate balance of cartilage homeostasis. Furthermore, novel Met‐mediated mechanisms governing cartilage senescence were identified through mRNA sequencing and network pharmacology analysis. Specifically, we identified inducible nitric oxide synthase (iNOS) as a protein potentially influenced by Met in senescent chondrocytes.

iNOS (NOSII or NOS2) is an exceptionally productive Ca^+^‐independent NOS.^[^ ^52^ ^]^ Its expression can be provoked in a diverse array of cells and tissues by cytokines and other stimulating agents.^[^ ^53^ ^]^ Once induced, iNOS persistently generates NO until the enzyme undergoes degradation.^[^ ^54^ ^]^ Substantial amounts of NO produced by iNOS exhibit advantageous properties, such as antitumoral, antiviral, antiparasitic and microbicidal actions.^[^ ^55^ ^]^ However, unwarranted iNOS induction can yield detrimental consequences and appears to be implicated in the pathophysiology of various human diseases including asthma, arthritis, multiple sclerosis, colitis, neurodegenerative diseases and tumor development et al.^[^ ^56^, ^57^ ^]^ The influence of NO on cellular survival or demise seems to be intricately connected to its biochemical impact on proteins via post‐translational modifications.^[^ ^58^ ^]^ Given its inherent instability, NO predominantly interacts with the superoxide anion, resulting in the formation of peroxynitrite (ONOO‐), a compound endowed with formidable oxidative characteristics.^[^ ^59^ ^]^ ONOO‐ is recognized as a consequential biological stimulator owing to its direct engagement with proteins via *S*‐nitrosylation.^[^ ^60^ ^]^ Within this investigation, we have unearthed that the administration of Met@SBHA diminished the manifestation of iNOS in senescent chondrocytes, thereby exerting a supplementary inhibitory effect on NO production. Moreover, we have furnished corroborative evidence indicating that the suppression of iNOS and NO expression by Met@SBHA may additionally decrease the generation of ONOO‐. These findings suggested that Met@SBHA may exert regulatory control over *S*‐nitrosylation, thereby inhibiting chondrocyte senescence. Intriguingly, evidence suggests that ONOO‐ might directly modulate P53 via *S*‐nitrosylation, consequently impacting the expression of this crucial protein.^[^ ^61^ ^]^ P53, an age‐related gene, governs apoptosis and cell cycle arrest in cells harboring DNA damage.^[^ ^62^ ^]^ Our findings demonstrate that the suppression of ONOO‐ leads to the downregulation of P53‐induced senescence. Additionally, in order to evaluate the influence of Met@SBHA on the *S*‐nitrosylation of P53, we assessed the expression of *S*‐nitrosylated P53 protein. Our results revealed that Met@SBHA effectively mitigates the *S*‐nitrosylation of P53. This novel discovery pertaining to the involvement of Met@SBHA in iNOS/ONOO‐/P53‐mediated senescence holds great promise for the therapeutic management of age‐related OA.

In addition, we have further explored the relevant assays concerning the mechanotransduction signaling pathway. In our aforementioned investigation of the biochemical signaling pathway, iNOS has been identified as the target protein of Met. iNOS is a proinflammatory factor,^[^ ^63^ ^]^ and our findings support this notion. We have observed elevated levels of IL‐1 and IL‐6 in senescent chondrocytes. Interestingly, IL‐1 and Piezo1 (a mechanically sensitive ion channel) establish a positive feedback loop during the onset of OA. This implies that inflammatory signaling can enhance Piezo1 mechanotransduction in articular chondrocytes as a pathogenic feed‐forward mechanism,^[^ ^64^ ^]^ while Piezo1 promotes the production of SASP factors, particularly in IL‐1.^[^ ^65^ ^]^ Furthermore, targeted suppression of Piezo1 in chondrocytes shows promise in alleviating OA pathogenesis.^[^ ^66^ ^]^ Our results also indicate that Piezo1 expression is upregulated in senescent chondrocytes, whereas Met@SBHA reduces Piezo1 expression and mitigates chondrocyte senescence. Additionally, Piezo1 functions as a Ca^2+^‐permeable ion channel, facilitating calcium influx in chondrocytes, thereby promoting cartilage degeneration.^[^ ^67^ ^]^ We have further examined the Ca^2+^ levels in senescent chondrocytes and have discovered a significant increase in Ca^2+^ levels in these cells, which is effectively inhibited by Met@SBHA.

## 4 Conclusion

In this study, with a primary emphasis on the pathogenesis of age‐related OA encompassing the senescence of cartilage and diminution of lubrication, we have innovatively devised and synthesized injectable Met@SBHA microspheres, which possess a dual functionality as both a biomimetic lubrication coating and a vehicle for Met delivery. mRNA‐Seq, network pharmacology, and in vitro*/*vivo assays were utilized to unravel the therapeutic effectiveness and underlying mechanism of Met@SBHA in alleviating age‐associated OA. Our findings revealed that Met@SBHA exhibited the capacity to counteract chondrocyte senescence and maintain the metabolic homeostasis of chondrocytes through the modulation of iNOS/ONOO‐/P53 axis. In summary, the functionalized microspheres presented in this study hold great promise as a potential approach to attenuate the degenerative alterations associated with age‐related OA.

## 5 Experimental Section

### Materials/Reagents

Di‐tert‐butyl dicarbonate, triethylamine, *N*, *N*‐dimethylethylenediamine, 1,3‐propanesulfonic acid lactone, acetonitrile, methacrylic anhydride, 4‐(4,6‐dimethoxy‐1,3,5‐triazin‐2‐yl)−4‐methylmorpholino chloride (DMTMM), trifluoroacetic acid (TFA), ion‐exchange resin (Amberlyst A26, in its hydroxide form), diethyl ether, *N*, *N*‐dimethylformamide (DMF), anhydrous magnesium sulfate, anhydrous ethanol, and dichloromethane (DCM) were obtained from McLean Biochemistry Co. Span 80 and HA were purchased from Aladdin Bio‐Chem Technology Co., Ltd. 2‐Hydroxy‐4′‐(2‐hydroxyethoxy)‐2‐methylpropiophenone (Irgacure 2959) and Lipopolysaccharide (LPS) were purchased from Sigma‐Aldrich Biochemical. The ATDC5 chondrocyte cell line were obtained from Merck KGaA. DMEM, fetal bovine serum, penicillin and streptomycin, 0.25% trypsin for cell culture were from Gibico. Calcein‐AM/propidium iodide (PI) kit, CCK8 detection kit, Cell Cycle and Apoptosis Analysis Kit, Senescence β‐Galactosidase Staining Kit, and NO assay kit were purchased from Beyotime Biochemical. The fluorescent probes Phalloidin‐TRITC, 5‐Ethynyl‐2′‐deoxyuridine (EdU) kit, Hieff NGS Ultima Dual‐mode mRNA Library Prep Kit were purchased from YEASEN Biochemical. RNA extraction kit was purchased from AG Biochemical. Pierce *S*‐nitrosylation Western blot kits were obtained from Thermo Fisher Scientific. Hematoxylin Eosin (H&E) taining Kit, Masson's stain kit, Safranin O‐fast Green cartilage staining kit, and Alcian blue staining kit were purchased from Solarbio Biochemical. The immunofluorescence staining primary antibodies were purchased from Proteintech Biochemical, including P53, P21, COL2A1, MMP13, Piezo1 and iNOS antibodies. The secondary antibodies, including Alexa Fluor 488‐ and 594‐conjugated were purchased from Thermo Fisher Scientific. Fluoroshield mounting medium with DAPI and neutral balsam were obtained from Sigma and Sanggon Biotech.

### Synthesis of SB‐NH_2_

The SB‐NH_2_ was synthesized by the published literature.^[^ ^68^ ^]^ A solution comprising of *N*,*N*‐dimethylethylenediamine (4.04 g, 45.8 mmol) in anhydrous ethyl alcohol (150 mL) was prepared at 0 °C. Di‐tert‐butyl dicarbonate (10.0 g, 45.8 mmol) and triethylamine (12.8 mL, 91.6 mmol) were then added drop by drop over a period of 0.5 h. The mixture was stirred for 1 h at 0 °C, followed by 18 h at room temperature. The white precipitate was filtered, and the filtrate was evaporated to yield a residue. The residue was dissolved in dichloromethane (150 mL) and washed with water. Subsequently, the magnesium sulfate was employed to dry it and then solution was evaporated to obtain *N*, *N*‐dimethyl‐2‐((pivaloyloxy) amino) ethan‐1‐amine.

A solution containing (7.52 g, 40.0 mmol) of N, N‐dimethyl‐2‐((pivaloyloxy)amino) ethan‐1‐amine, (4.9 g, 40.0 mmol) of 1,3‐propanesultone, and 150 mL of acetonitrile was combined. The mixture was stirred at 40 °C under a nitrogen atmosphere for 48 h. Subsequently, the solvent was removed using a rotary evaporator. The product was obtained as a white powder through precipitation with anhydrous diethyl ether, followed by washing with additional anhydrous diethyl ether.

The obtained product (10.0 g) was subjected to treatment with a mixture of 20 mL TFA and 20 mL dichloromethane for 12 h at RT. The solution was subsequently concentrated by a rotary evaporator, precipitated in anhydrous diethyl ether, and redissolved in deionized water. The solution was subjected to neutralization by an ion‐exchange resin (Amberlyst A26, in its hydroxide form). The remaining substance was subsequently subjected to lyophilization using a freeze dryer, culminating in the formation of the intended product (SB‐NH_2_).

### Synthesis of SBHA

HAMA were synthesized according to previously reported methods.^[^ ^69^ ^]^ A reaction was initiated with a 2 wt% concentration of HA (Mw = 100 kDa; China) and methacrylic anhydride (Aladdin, China) at alkaline condition (pH = 8.0). The solution was continuously agitated for 24 h at 0 °C. The product was dialysis for 3 days. Subsequently, the purified product was freeze‐dried.

A solution of 200 mg of HAMA was dissolving in 10 mL of distilled water. Subsequently, 180 mg of DMTMM was dissolved in the HAMA solution. Second, 200 mg of SB‐NH_2_ was then mixed with the HAMA solution and the pH was adjusted to 6.5. The resulting mixture was stirred 24 h and dialysis for 3 days. The supernatant liquid that was subsequently freeze dried.

### Characterization and Preparation of Hydrogel Microspheres

Briefly, 5% (w/v) HAMA or SBHA solution in phosphate buffer saline (PBS) supplemented with 0.5% Irgacure 2959 served as the internal aqueous phase, while the continuous oil phase was composed of paraffin oil. Span 80 was used as the surfactant to stabilize the droplets. The internal and continuous phases were slowly injected into the microfluidic device at the flow rates of 1 mL h^−1^ and 6 mL h^−1^, respectively. The hydrogel droplets were subsequently transferred to the disc‐shaped platform, where they underwent solidification through photo‐crosslinking under UV irradiation at a wavelength of 365 nm (6.9 mW cm^−2^) for a duration of 20 s. Then, the photopolymerized microspheres were collected into each microtube, and washed repeatedly with acetone and 75% alcohol to remove the oil and surfactant, followed by washing with fresh PBS every 4 h for 24 h for further purification. Finally, the hydrogel microspheres were examined by microscope (Olympus, USA). The morphology of microspheres was examined by High‐resolution SEM (Thermo Fisher Scientific Apreo S, USA) after freeze‐drying.

Microsphere size and zeta potential were determined by a Brookhaven 90Plus DLS instrument (Brookhaven, USA). HA, HAMA, SBHA were used for ^1^H‐NMR analysis on a Bruker 400 MHz AVANCE III NMR spectrometer. The chemical composition of the samples was characterized by X‐ray photoelectron spectroscopy (XPS, PHI Quantera II, Ulvac‐Phi Inc., Japan) and Fourier transform infrared spectroscopy (FT‐IR, Nicolet IS50, Thermo Fisher Scientific, MA, United States).

The morphological detection and mechanical properties of microspheres were measured using AFM (Bruker Dimension ICON, US). The sample concentration was adjusted to ≈0.1 mg mL^−1^ using aqueous dispersion, and the solution was vortexed for 10 s to disperse the solution on the wafer. The selected silicon cantilever was Bruker PTESPA‐525, with a tip radius of curvature of ≈10 nm, was used to determine the specific Kc value of the probe by thermal modulation prior to the measurement. The 2D and 3D morphology of the samples were measured in the PeakForce Tapping mode of the ICON‐type AFM, and the force‐displacement curves (referred to as force curves) of the samples were recorded (with a cantilever spring constant of k of 110.56 pN nm^−1^, a probe speed of 2 µm s^−1^, and a force trigger of 5 nN). All tests were completed at 25 °C and 40% relative humidity. The data from the fitted force curves were analyzed by NanoScope Analysis software, and the compressive Young's modulus of the samples was calculated from the elastomechanical contact model.

### Tribological Test

The tribological tests were performed by a universal materials tester (UMT‐3, Bruker Nano Inc., Germany) in a reciprocating mode at room temperature for 600 cycles. All the tribological experiments were completed with pin‐on‐disk friction pairs, using polytetrafluorethylene (PTFE) pin (diameter of contact surface: 5 mm) as the upper sample and silicon wafer as the lower sample.^[^ ^70^, ^71^, ^72^ ^]^ Specifically, 20 drops of different microspheres/PBS solutions (5 mg mL^−1^) were dropped into the contact area as the lubricant before each experiment, with PBS as the control group. The oscillation amplitude was 4 mm, the sliding frequency was 1 Hz, and the normal load was 12 N. Each test was repeated at least three times to ensure data validity.

### Drug Release Test and Degradation Test In Vitro

Met solutions with a series of concentration gradients were prepared in PBS (pH = 7.4) and the absorbance was recorded in the range of 200 – 500 nm using a NanoDrop One microvolume UV–vis spectrophotometer (Thermofisher Scientific, USA) to obtain a full wavelength scan of the UV spectrum, based on which the calibration curve of Met was obtained (Absorbance at 233 nm). The tube containing 5 mL of Met@SBHA suspension was placed on a thermostatic shaker (37 °C, 80 rpm). The supernatant was collected for UV absorbance measurement at certain time points. After detection, the solution was returned to the tube and shaken at a constant temperature until the microspheres were completely degraded. Degradation of Met@SBHA was performed in PBS solution of collagenase, which mimics the physiological environment in vivo. 30 mL of Met@SBHA was immersed in 0.2% collagenase type II (pH 7.4) and digested at 37 °C and 80 rpm with agitation. The collagenase solution was replenished every 2 days to maintain enzyme activity. Degradation of Met@SBHA was analyzed by measuring the residual weight at fixed times and comparing it with the initial weight.

### Cell Culture and Treatments

Primary chondrocytes were obtained from the cartilage cap tissue of the knee joint of 1‐month‐old SD rats. The trimmed cartilage fragments were digested with 0.25% trypsin for 15 min and 0.2% collagenase Type II for 4 h in an oscillating water bath at 37 °C. Chondrocytes suspension were filtered through 100 µm nylon filters, and inoculated at density of 1.5 × 10^5^ cm^2^. Primary chondrocytes from the second or third generation were used for this series of experiments. The primary chondrocytes and ATDC5 were incubated with DMEM, 10% FBS, 1% penicillin, and streptomycin in the incubator, and insulin (10 µg mL^−1^) was induced into chondrocytes for subsequent experiments.

Based on CCK8 experiments and RT‐qPCR detection of the expression levels of senescence indicators P21 and P53 (Figure S15, Supporting Information), we selected 40 µg mL^−1^ LPS concentration to stimulate chondrocytes for 48 h to construct a senescence mode^[^ ^73^, ^74^, ^75^ ^]^ and then co‐cultured with the prepared microspheres material. 1 mM has been identified as the optimal metformin dosage within the microspheres.

### Biocompatibility

Chondrocytes were co‐cultured with the material for 24 h and labeling by the Calcein/PI dye for 30 min at dark environment. The live/dead cells were captured through a fluorescence microscope (Nikon N31373). The obtained images were then quantified and statistically analyzed using ImageJ software (version 1.53a).

Chondrocytes were inoculated into 24‐well plates at a density of 1 × 10^4^ per well and subsequently co‐cultivated with sterilized microspheres for 24 h. Following fixation in 4% paraformaldehyde for 15 min, the cells were then subjected to permeabilization by treatment with 0.5% Triton X‐100 for 15 min. Subsequently, a 30 min incubation period with a 1% BSA solution, diluted with Phalloidin‐TRITC dye, was carried out. Prior to fluorescence microscopy imaging, the cell nuclei were retained with DAPI.

### Cell Proliferation and Cell Cycle Assay

Chondrocytes proliferation viability was assessed with the CCK‐8 kit, following operational guidelines. Briefly, cells were seeded in 96‐well plates at a density of 3000 cells per well. Subsequently, 10 µL of CCK‐8 solution was added to each well and incubated for 2 h. The absorbance was measured at a wavelength of 450 nm using the Infinite M200 PRO plate reader (Tecan, Switzerland).

Chondrocyte proliferation was assessed by a Yefluor 488 Edu Cell Imaging Kit. Initially, cells were labeled with a 10 µM Edu working solution for 2 h. Subsequently, cells were fixed in 4% paraformaldehyde for 30 min, neutralized with 50 µL of 2 mg ml^−1^ glycine for 5 min, washed twice with 3% BSA in PBS, and incubated with 0.5% Triton X‐100 for 10 min. Finally, the cells were incubated with 100 mL of Click‐iT reaction mixture and Hoechst (nuclear stain) for 30 min at 37 °C in dark and then observed under a fluorescence microscope (NIKON).

The Cell cycle and apoptosis analysis kit was used for cell cycle analysis. Cells were treated by 0.25% trypsin, and followed by two washes with PBS. Subsequently, the cells were fixed in a 70% pre‐cooled ethanol solution at 4 °C overnight. The following day, the fixed cells were washed twice with PBS. The cells were then resuspended in 535 µL of propidium iodide (PI) solution (comprising 500 µL of dye buffer, 25 µL of 20 × PI stain, and 10 µL of 50 × RNase A) and incubated at 37 °C for 30 min, while ensuring avoidance of light. The cell cycle was subsequently detected at an excitation wavelength of 488 nm using Cytoflex (Beckman Coulter).

### SA‐β Galactosidase Staining

The Senescence β‐galactosidase staining kit was employed to conduct the β‐galactosidase staining, following the guidelines provided by the manufacturer. In brief, 5 × 10^5^ cells were seeded onto 6‐well plates. Subsequently, the cells were fixed using 4% paraformaldehyde for a duration of 20 min, followed by staining with the working solution at 37 °C overnight. On the subsequent day, the cells were rinsed thrice with PBS and observed under a microscope. The senescence rate was determined by quantifying the proportion of positive cells.

### Evaluate Chondrocyte Degradation

To assess the degradation of chondrocytes, the Alcian blue and Safranin O‐fast green staining technique was employed. For Alcian blue staining, the cultured chondrocytes were immobilized in 80% methanol for 20 min, followed by incubation with a solution of 0.1% HCl‐alcian blue for 2 h. Any excess stain was subsequently rinsed away. For Safranin O staining, cells were fixed and stained for 5 min, followed by washing and differentiation in an acidic differentiation solution for 15 s. Subsequently, the stained chondrocytes were captured under an optical microscope for documentation.

### Chondrocytes Immunofluorescence Staining

The cultured chondrocytes were initially fixed using a 4% paraformaldehyde solution in phosphate‐buffered saline. Subsequently, they were permeabilized with 0.25% Triton‐X and subjected to a blocking procedure using a solution composed of 2% glycine, 2% bovine serum albumin, and 5% fetal bovine serum, along with 50 mmol L^−1^ NH_4_Cl in phosphate‐buffered saline for a duration of 1 h. Following this, the cells were incubated with primary antibodies MMP13 (1:500), COL2A1 (1:500), P21 (1:500), P53 (1:500), and iNOS (1:500) for a period of 2 h, followed by incubation with corresponding secondary antibodies conjugated to various fluorescent agents. To facilitate nuclear staining, DAPI was introduced to the cells and allowed to incubate at room temperature for 10 min. After thorough washing, the coverslips were air‐dried, mounted, and prepared for fluorescence microscopy. Images were captured using a Nikon Eclipse Ti‐S inverted microscope equipped with NIS‐BR Microscope Imaging Software.

### RNA Extraction and Real‐Time Quantitative RT‐PCR

Total RNA was extracted from cultured cells utilizing the Trizol reagent in accordance with the guidelines provided by the manufacturer. The optical density (OD) of the total RNA was measured, and an OD_260_/OD_280_ > 1.8 was employed for subsequent quantification through reverse transcription polymerase chain reaction (RT‐PCR). Subsequently, the PrimeScript TM reagent kit with gDNA Eraser was employed to convert 1 µg of total RNA into complementary DNA (cDNA) within a 20 µL volume. Quantitative real‐time PCR was conducted using the Quantitative Real‐time PCR Kit. All primers were meticulously designed and synthesized by Yeasen Biotechnology. The outcomes were standardized using GAPDH as an internal control. The specific primers employed for mRNA amplification were provided (Table S3, Supporting Information)

### *S*‐Nitrosylated Protein Detection

Protein‐SNO was identified using the *S*‐nitrosylated protein detection kit with minor adjustments. Briefly, in accordance with the manufacturer's instructions, cells were lysed in a lysate buffer containing a thiol‐specific methylthiolating reagent (methyl methanethiosulfonate, MMTS) to inhibit the activity of free reactive thiol groups. After centrifugation, the supernatants were transferred to a new polypropylene tube and subjected to acetone precipitation to eliminate any remaining free MMTS. Buffers containing reducing and labeling agents (biotin‐HPDP and ascorbic acid sodium salt) were used to resuspend the resulting pellets, thereby converting the SNO site into a biotin‐tag. Acetone‐washed precipitation was performed once again to remove any residual free biotin‐HPDP, and the pellets were stored for immunoprecipitation‐based analysis of SNO. The detection of *S*‐nitrosylated P53 protein was carried out through SDS‐PAGE of labeled protein and subsequent Western blotting analysis.

### Western Blot

Following co‐incubation of the materials, cells were treated with RIPA lysis buffer containing a blend of protease and phosphatase inhibitors (Roche, Switzerland) on ice for 30 min. Protein concentration was determined and protein samples were loaded onto SDS gel, electrophoretically separated for 70 min, and subsequently transferred to a PVDF membrane for 1.5 h. The bands were blocked using milk and then incubated overnight with the primary antibody. After washes with TBST, the bands were incubated with the secondary antibodies IgG HRP at RT for 60 min. Following ECL color development, the results were analyzed using Image J software to calculate the grey value of the protein bands.

### Peroxynitrite Anion (ONOO‐) Detection

Cells were seeded in the chamber at a density of 2 × 10^4^ cells per well. After 24 h, the cells were subjected to specific treatments as per the experimental groups. Subsequently, the cells were fixed with a 4% solution of PFA for 10 min, followed by incubation with the 10‐fold dilution of BBoxiProbe O71 fluorescent probe working solution at a temperature of 37 °C for 60 min. The samples were then observed under the microscope after washed, and the resulting images were analyzed by Image J software.

### Intracellular Ca^2+^ Levels Detection

Intracellular Ca^2+^ levels were detected using the visible light‐excited fluorescent probe Rhod‐2. Briefly, the Rhod‐2/AM master mix was diluted into a 5 µM working solution, and was co‐cultured with treated cells at 37 °C for 30 min Cells were washed three times to remove uncoupled dye and incubated for a further 30 min to allow complete de‐esterification of intracellular AM ester in a 37 °C incubator. Nuclei were labelled with Hoechst at 37 °C for 15 min in dark before observation under fluorescence microscope. To simulate the mechanical stress conditions, cells were cultured under shaking conditions (37 °C, saturated humidity, 60 r min^−1^).

### Measurement of Nitric Oxide

The measurement of NO production was conducted utilizing a NO assay kit in accordance with the provided guidelines. Briefly, 50 µL of supernatant from each well was transferred to a separate 96‐well plate and combined with an equal volume of griess reagents, allowing for a 10 min incubation period. The absorbance values at 540 nm were subsequently determined utilizing a microplate reader. The concentrations of NO were then calculated based on the established standard curve of NaNO_2_.

### RNA‐Sequencing Analysis

Total cellular RNA was extracted using Trizol reagent. The extracted RNA samples were then subjected to RNase‐free agarose gel electrophoresis to assess the integrity of the nucleic acid samples. The quality of the RNA was further evaluated using an Agilent 2100 Bioanalyzer (Agilent Technologies, Palo Alto, CA, USA), following the standard operating procedures provided by the manufacturer.

For subsequent RNA sequencing library construction, the Hieff NGS Ultima Dual‐mode mRNA Library Prep Kit (12309ES, Yeasen, China) was employed. First‐strand cDNA synthesis was carried out immediately after mRNA enrichment and fragmentation using Oligo (dT) magnetic beads. The second‐strand cDNA was synthesized using DNA polymerase I, RNase H, dNTP, and buffer. Purified or length‐selected junction products were then enriched through PCR amplification and subsequently sequenced on the Illumina HiseqTM 2500/4000 by Gene Denovo Biotechnology Co., Ltd.

The functions and pathways of the differentially expressed genes were analyzed using the GO database (http://www.geneontology.org/), KEGG database (http://www.kegg.jp/KEGG/), and Reactome database (https://reactome.org/).

### Metformin Target Prediction and OA‐Related Target Screening

The cheminformatics data for Met was obtained from the PubChem database (https://pubchem.ncbi.nlm.nih.gov/), while the prediction of 84 pharmacological targets was acquired through the utilization of the accessible online the Swiss Target Prediction Database (http://swisstargetprediction.ch/). Simultaneously, the gene expression data for OA was gathered from GeneCards platform (https://www.genecards.org/). The intersection of Met targets and OA‐related genes was visually represented as a Venn diagram by the “VennDiagram” package in R version 4.2.3. Subsequently, the aforementioned intersecting genes were inputted into Cytoscape software, and the “Bisogenet” package within the software was employed to conduct protein‐protein interaction (PPI) analysis, resulting in the generation of a PPI network diagram.

### GO and KEGG Pathway Enrichment Analyses

The overlapping target genes underwent Gene Ontology (GO) and Kyoto Encyclopedia of Genes and Genomes (KEGG) enrichment analyses utilizing the “colorspace”, “stringi”, and “ggplot2” packages in the R programming language. For this analysis, the “DOSE”, “clusterProfiler”, and “enrichplot” packages from the BiocManager database (http://www.bioconductor.org/) were employed. The filtering criteria were set to “hsa” for the organization, with a “P value Cutoff” and “Q value Cutoff” of 0.05. The outcomes of the GO enrichment analysis and KEGG pathway enrichment analysis for cellular component (CC), biological process (BP), and molecular function (MF) were visually presented using bubble charts. The overlapping target genes were analyzed for GO and KEGG enrichment using the “clusterProfiler” and “ggplot2” packages in R. A significance threshold of 0.05 was applied for both the P‐value and Q‐value. The results, depicted as bubble charts, showcased the enriched biological processes in terms of cellular component (CC), biological process (BP), and molecular function (MF) for both GO and KEGG.

### Molecular Docking

Initially, the cheminformatics data of Met was obtained from the PubChem database (https://pubchem.ncbi.nlm.nih.gov/), encompassing its chemical nomenclature, molecular formula, CAS number, PubChem CID, canonical SMILES, and SDF files. The SDF file containing the 3D structure of Met was subsequently converted to a Mol2 file using the versatile Open Babel software (https://openbabel.org/wiki/Main_Page). As for the protein targets, their respective 3D structures were procured from the PDB database (http://www.rcsb.org/). The AutoDockTools software (version 1.5.6, https://autodocksuite.scripps.edu/) was employed to process these PDB files, eliminating water molecules, introducing hydrogen atoms, and assigning Kollman charges. Ultimately, the data were saved as PDBQT files. The GridBox parameters were meticulously determined based on the binding region of the protein receptor and the original ligand. To assess the affinity (expressed in kcal mol^−1^) of all potential key targets for Met, the AutoDock Vina software (version 1.1.2, http://vina.scripps.edu/) was utilized. Generally, a lower affinity value signifies a stronger binding between the small molecule and the receptor. To visually depict the Met‐target protein interaction, the proficient PyMOL software (version 2.5.4, https://pymol.org/) was employed, and the resulting visualizations were saved as PNG images.

The SDF format was converted to Mol2 format using Open Babel (https://openbabel.org/wiki/Main_Page). The 3D structures of proteins were obtained from the PDB database (http://www.rcsb.org/). AutoDockTools (version 1.5.6) and PyMOL (version 2.5.4) software were employed for molecular docking and figure generation. Generally, a lower affinity value in the docking results indicates a more stable binding between the protein and ligand, reflecting superior binding energy in protein‐ligand interactions.

### Animal Model and Treatment

The aged C57BL/6J mice (male, 17 months of age) were procured from Southern Medical University, adhering to the guidelines set forth by the Experimental Animal Ethics Committee of Southern Medical University (Ethics number: SMUL202403053). The animal experiments were conducted in accordance with the esteemed National Research Council's Guide for Care and Use of Laboratory Animals. Adequate provisions of food and water were made available to the animals. The mice were housed in controlled barrier facilities, maintaining a 12 h light/dark cycle, with an ambient temperature ranging from 18 to 22 °C and a relative humidity of 50%−60%. For the microsphere's implantation treatment, briefly, after opening the joint capsule, the materials were intra‐articular injected to different groups: 17 months‐operated treated with physiological saline, Blank group‐operated treated with HAMA microspheres, Lubrication group‐operated treated with SBHA microspheres, Met@SBHA‐operated treated with Met@SBHA microspheres. Mice were given the intra‐articular injection using 10 µL Microliter Syringes. All mice were sacrificed at 8 weeks after the knee‐surgery.

### Evaluation of In Vivo Retention Time

Retention time was assessed by injecting FITC fluorescently labelled microspheres into mouse knee joints. FITC‐NH_2_ was synthesized according to a previous method as a means of labelling the microspheres.^[^ ^76^ ^]^ The fluorescence intensity was detected at the excitation wavelength of 488 nm and emission wavelength of 525 nm by IVIS spectrum system (Xenogen, USA) at different time points after joint cavity injection.

### Histological Evaluation

The knee joints samples were fixed in a 4% PFA for 24 h, followed by decalcification with a 10% ethylenediaminetetraacetic acid (EDTA) solution (pH 7.4) in a shaker at 37 °C and 80 rpm for 7 days. After gradual dehydration, the knee joints were embedded in paraffin. Histological staining was performed by making 4 µm incisions along the coronal plane. This included the utilization of the H&E staining kit, Masson's stain kit, Safranin O‐fast green cartilage staining kit, Alcian blue staining kit and senescence β‐Galactosidase staining kit. The thickness ratio of calcified cartilage (CC) to hyaline cartilage (HC) in the cartilage was calculated and scored according to the OARSI (Osteoarthritis Research Society International)‐modified Mankin criteria. Additionally, heart, liver, spleen, lungs, kidneys, and other organs from the experimental animals were also subjected to H&E staining. Blood counts, ALT/AST levels, and CREA‐S levels in mice from each experimental group were measured to evaluate the biosafety.

### Immunofluorescence and Immunohistochemistry Staining

The slide specimens were subjected to restoration in a Tris EDTA repair solution with a pH of 9, heated to a temperature of 75 °C for a duration of 45 min. Following this, a 3% H_2_O_2_ solution was applied at room temperature for 15 min to neutralize any remaining peroxidase activity. Subsequently, closure was achieved by treating the specimens with 5% goat serum at a temperature of 37 °C for a period of 30 min. After these meticulous steps, the specimens were meticulously washed three times with PBST and PBS. Next, 100 µL of dilutedprimary antibody was incubated overnight, and an appropriate secondary antibody was selected for subsequent incubation. Prior to the visualization of immunofluorescence, the nuclei were stained with DAPI. For the immunohistochemistry, a DAB chromogenic kit (Cat#ZLI‐9018, ZSGB‐BIO, China) was employed to facilitate the development of color, and the specimens was stained with hematoxylin before imaging.

### Microcomputed Tomography Analysis

The samples were meticulously scanned and reconstructed using the cutting‐edge technology of high‐resolution microcomputed tomography (CT) with the SkyScan 1172 machine. The resulting data was processed using CT reconstruction software, NRecon v1.6. For the visualization of 3D models and further analysis, the tools of CTAn v1.9 and µCTVol v2.0 were employed. The scanner parameters were set at a voltage of 50 kVp, a current of 200 µA, and a remarkable resolution of 9 µm per pixel. The region of interest selected for analysis encompassed the entire subchondral bone of the specimens. Key measurements, including Tb.Pf, BV/TV, Tb.N, and SBP.Th, were recorded and analyzed.

### Statistical Analysis

Statistical analyses were performed using BMI SPSS (version 21). Differences between two groups, assuming normal distribution, were analyzed using the independent t‐test. In cases of non‐normal distribution, the Mann‐Whitney test was employed. For comparisons among multiple groups, one‐way ANOVA was utilized, followed by pairwise comparison using the LSD method. Graphs were generated using GraphPad Prism 9. Statistical significance was denoted as *P < 0.05; **P < 0.01; ***P < 0.001; and ****P < 0.0001.

## Conflict of Interest

The authors declare no conflict of interest.

## Author Contributions

J.H., Y.L., and C.Z. contributed equally to this work. Z.C., H.W., J.W., D.G., and B.Y., conceived and designed the experiments. Z.C., H.W., J.H., and Y.L., performed and wrote the manuscript. J.H., Y.L., C.Z., analyzed the data. J.H., R.L., and H.L., Y.C., and prepared all the figures. All authors reviewed and agreed upon the manuscript.

## Supporting information

Supporting Information

## Data Availability

The data that support the findings of this study are available from the corresponding author upon reasonable request.
