# Supplementary material for: Zwitterion‐Lubricated Hydrogel Microspheres Encapsulated with Metformin Ameliorate Age‐Associated Osteoarthritis
Source: Adv Sci (Weinh). 2024 Jun 14;11(30):2402477. doi: 10.1002/advs.202402477 (PMC11321630; doi:10.1002/advs.202402477)
Supplement: Supplementary file 1 — Supporting Information [file ADVS-11-2402477-s001.pdf]

ADVANCED SCIENCE
Open Access

Supporting Information

for *Adv. Sci.*, DOI 10.1002/advs.202402477

Zwitterion-Lubricated Hydrogel Microspheres Encapsulated with Metformin Ameliorate Age-Associated Osteoarthritis

Jiahui Hou, Yanpeng Lin, Chencheng Zhu, Yupeng Chen, Rongmin Lin, Hancheng Lin, Dahai Liu, Daogang Guan, Bin Yu, Jun Wang, Hangtian Wu* and Zhuang Cui**

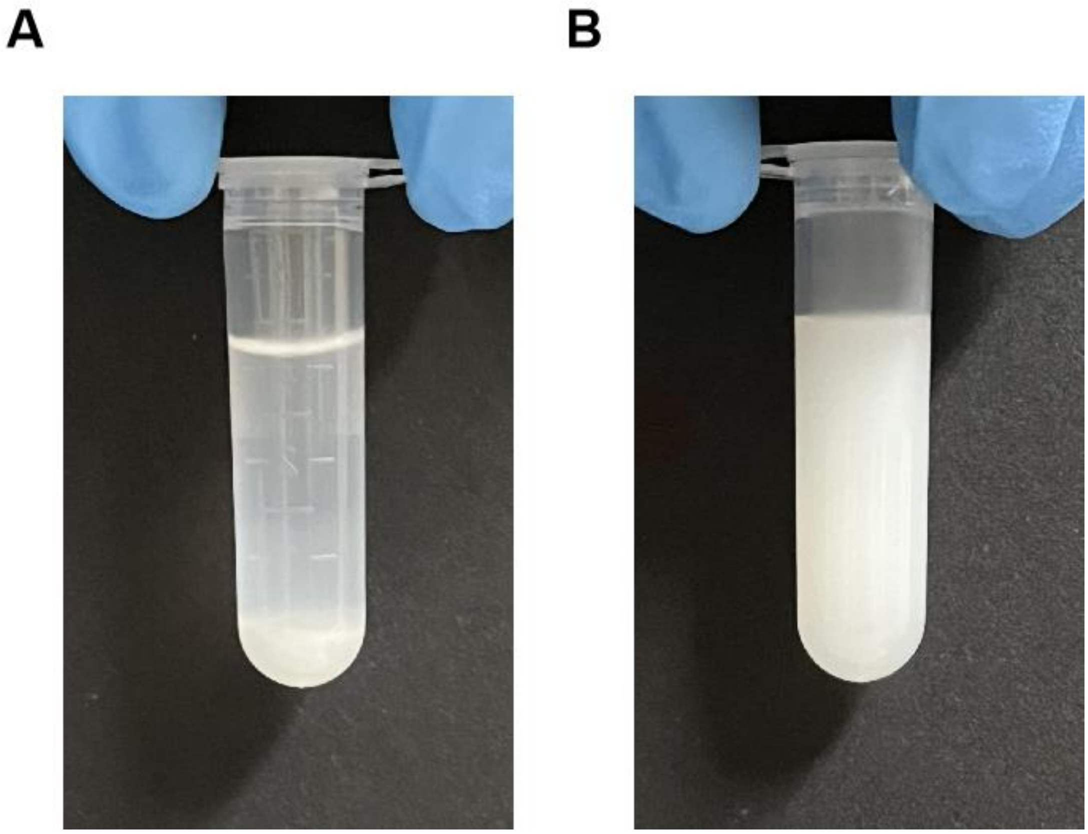

Figure S1. **A**) Precipitation state of hydrogel microspheres after standing. **B**) Translucent suspension of hydrogel microspheres after shaking.

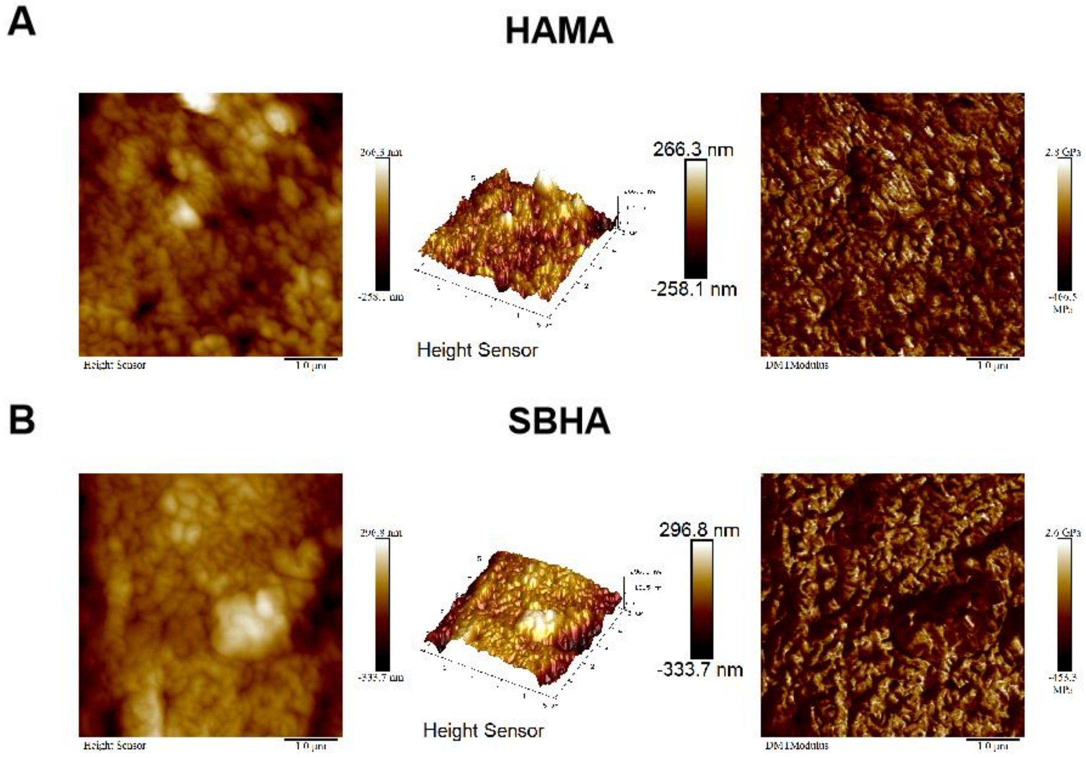

Figure S2. **A-B**) 2D and 3D morphology maps, phase diagrams of HAMA and SBHA microspheres.

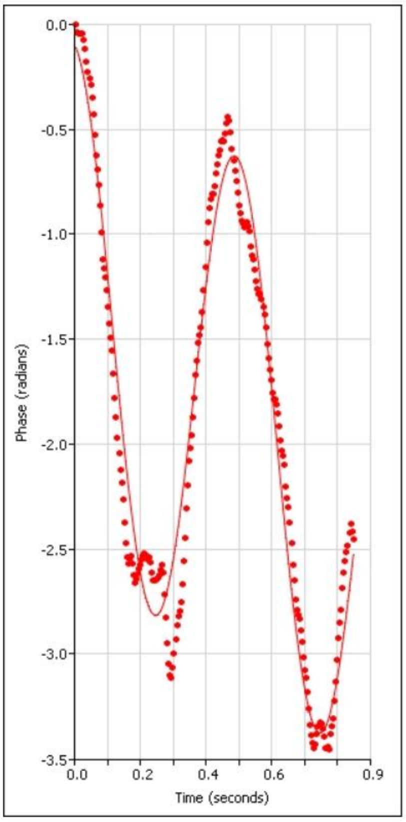

Figure S3. The zeta potential of Met@SBHA

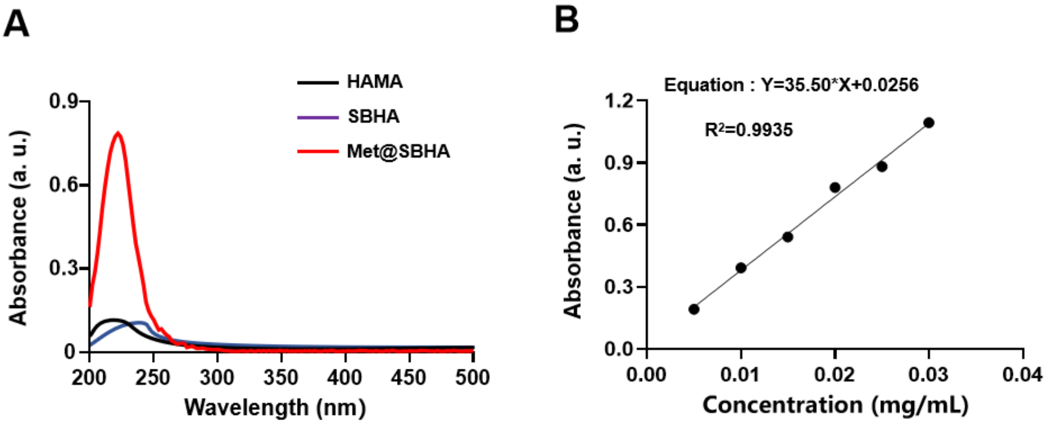

Figure S4. **A**) Full-wavelength scanning of the UV spectrum for HAMA, SBHA and Met@SBHA. **B**) UV absorbance calibration curve for metformin.

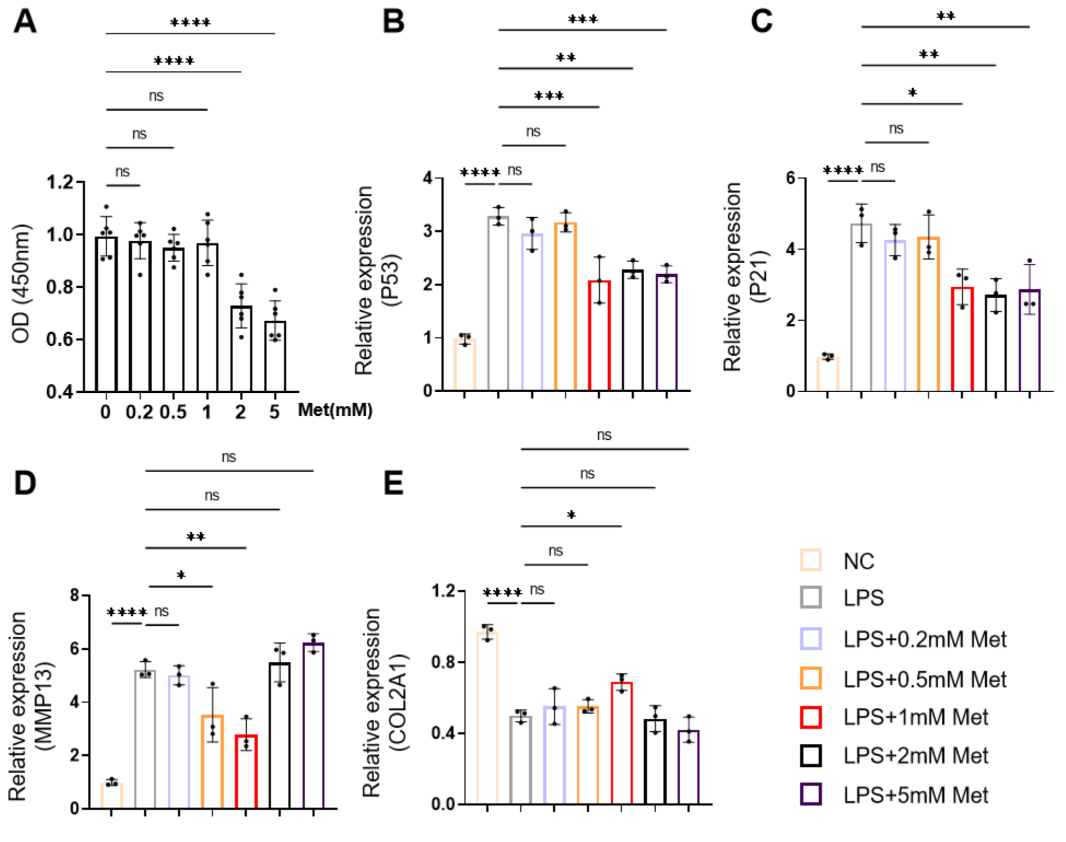

Figure S5. **A**) CCK8 assay to detect chondrocyte viability after co-culture with microspheres encapsulated with different concentration gradients of metformin. **B-E**) RT-qPCR detection relative mRNA expression levels of P53, P21, MMP13, and COL2A1 in chondrocytes co-cultured with microspheres encapsulated with different concentration gradients of metformin. Number of repetitions of all experiments $n \geq 3$. Data are presented as the mean ± SD. (*, $P < 0.05$; **, $P < 0.01$; ***, $P < 0.001$ and ****, $P < 0.0001$)

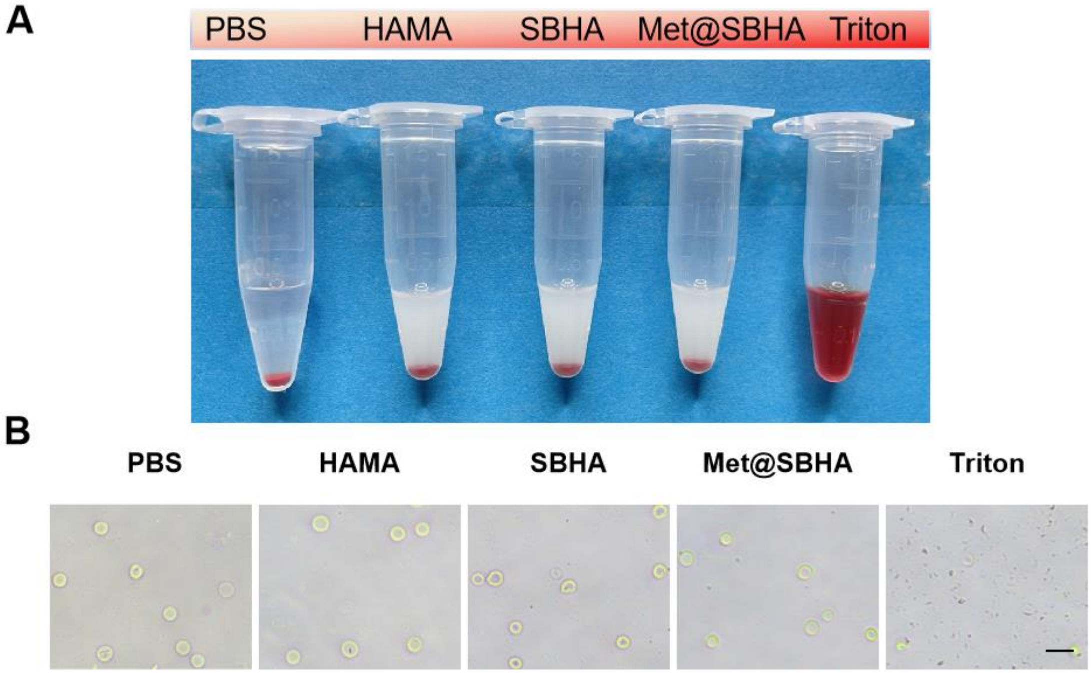

Figure S6. **A**) Hemolysis test of each hydrogel microsphere group. **B**) Microscopic observation of erythrocytes in each hydrogel microsphere group. Scale bars: 500 μm.

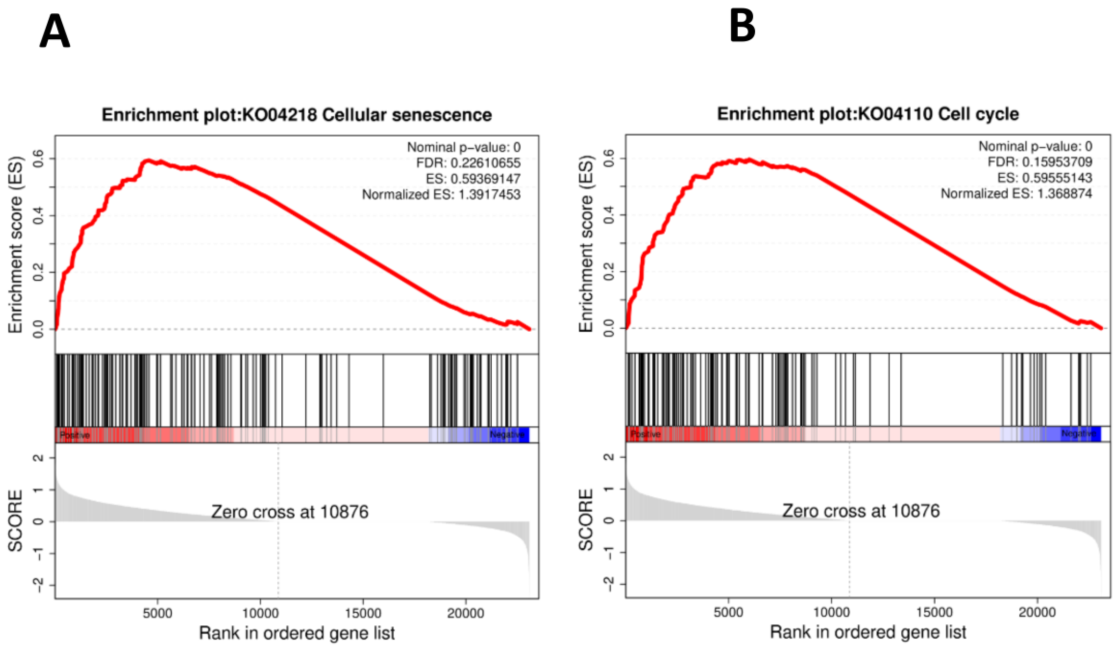

Figure S7. GSEA enrichment for analyzing cellular senescence and cell cycle, n=3.

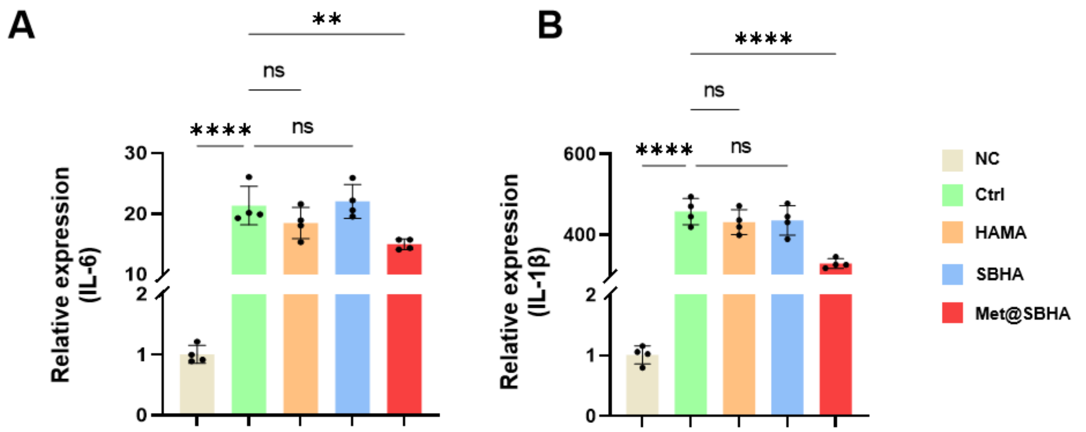

Figure S8. **A-B**) Relative RNA expression levels of the pro-inflammatory cytokines IL-1β and IL-6 associated with senescence-related secretory phenotypes. Number of repetitions of all experiments n ≥ 3. Data are presented as the mean ± SD. (*, $P < 0.05$; **, $P < 0.01$; ***, $P < 0.001$ and ****, $P < 0.0001$)

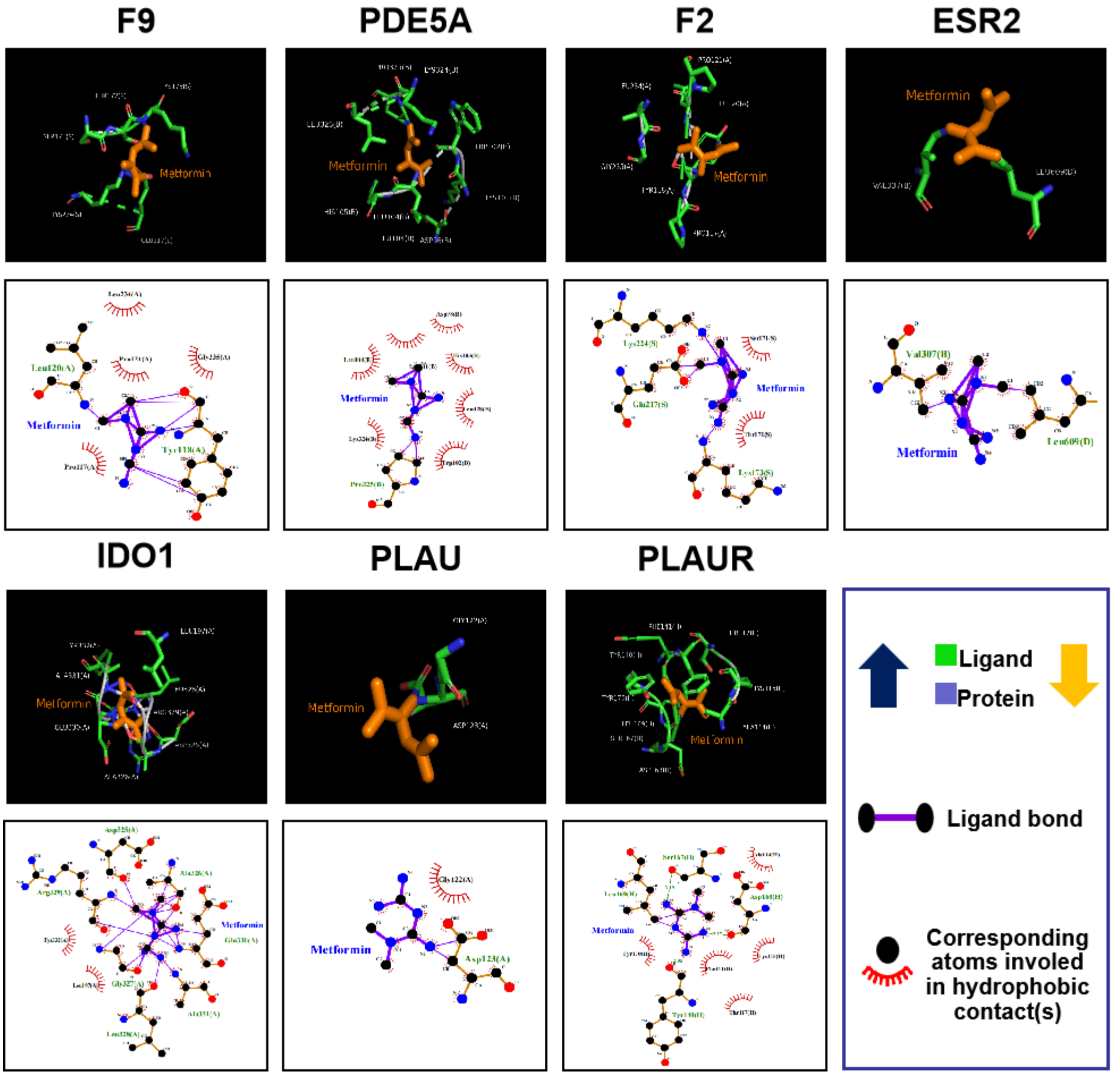

Figure S9. The binding site and site-site interactions of Met and F9, PDE5A, F2, ESR2, IDO1, PLAU, and PLAUR proteins.

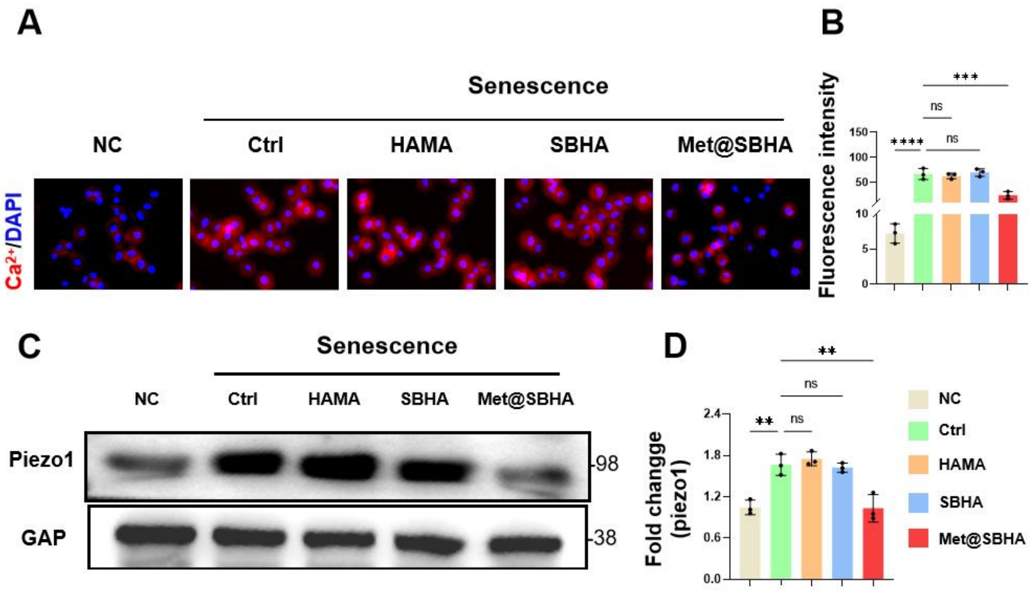

Figure S10. **A-B**) Detection of intracellular Ca^{2+} content by probe labelling. **C-D**) Representative immunoblot analysis and quantification of Piezo1 protein expression in treated chondrocytes. Scale bars: 500 µm. Number of repetitions of all experiments n ≥ 3. Data are presented as the mean ± SD. (*, P < 0.05; **, P < 0.01; ***, P < 0.001 and ****, P <0.0001)

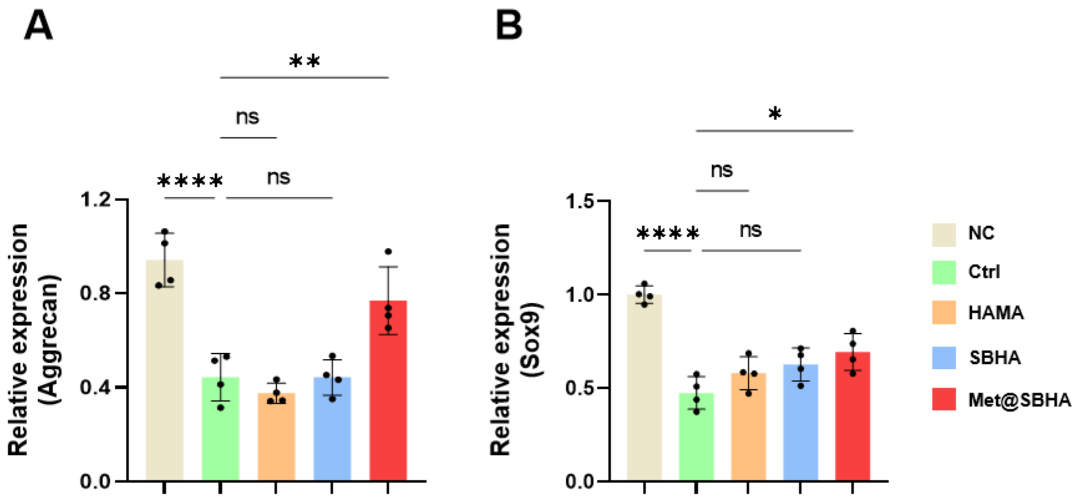

Figure S11. **A-B**) Relative RNA expression levels of Aggrecan and Sox9 in each group. Number of repetitions of all experiments n ≥ 3. Data are presented as the mean ± SD. (*, $P < 0.05$; **, $P < 0.01$; ***, $P < 0.001$ and ****, $P < 0.0001$)

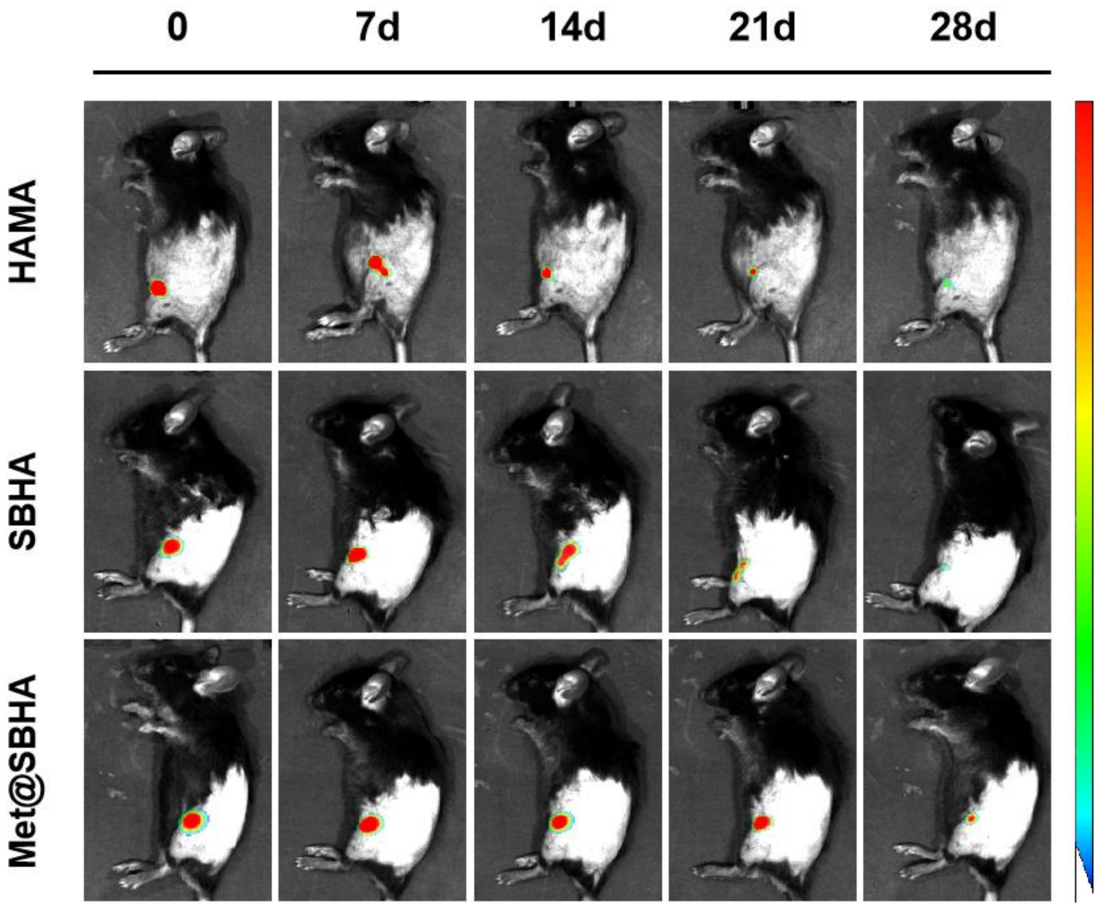

Figure S12. IVIS images of fluorescently labelled microspheres injected into the joint cavity at different time points

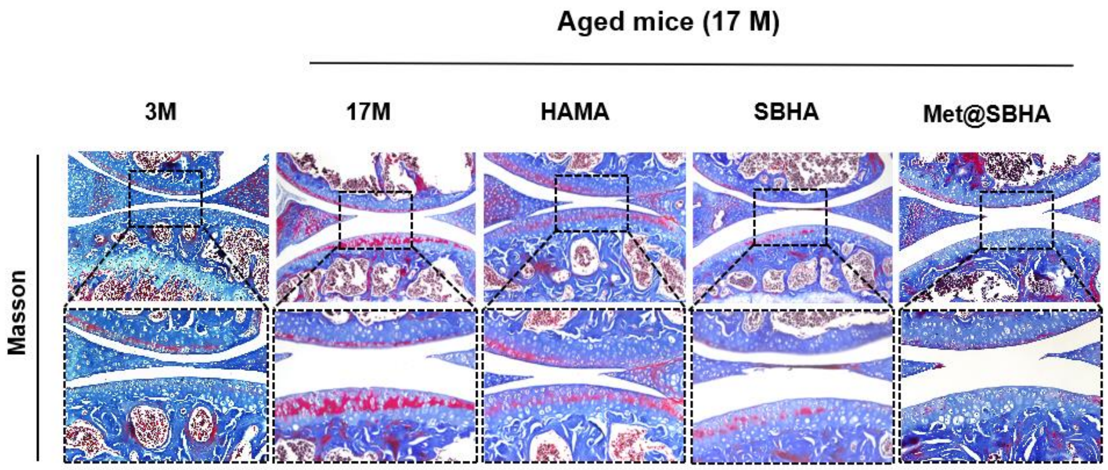

Figure S13. Representative images of Masson staining in 3M group, 17M group, 17M+HAMA group, 17M+SBHA group, and 17M+Met@SBHA group; the blue portion represents collagen, while the red is fibrillar staining. n=3. Scale bar: top 100 µm; bottom 50 µm.

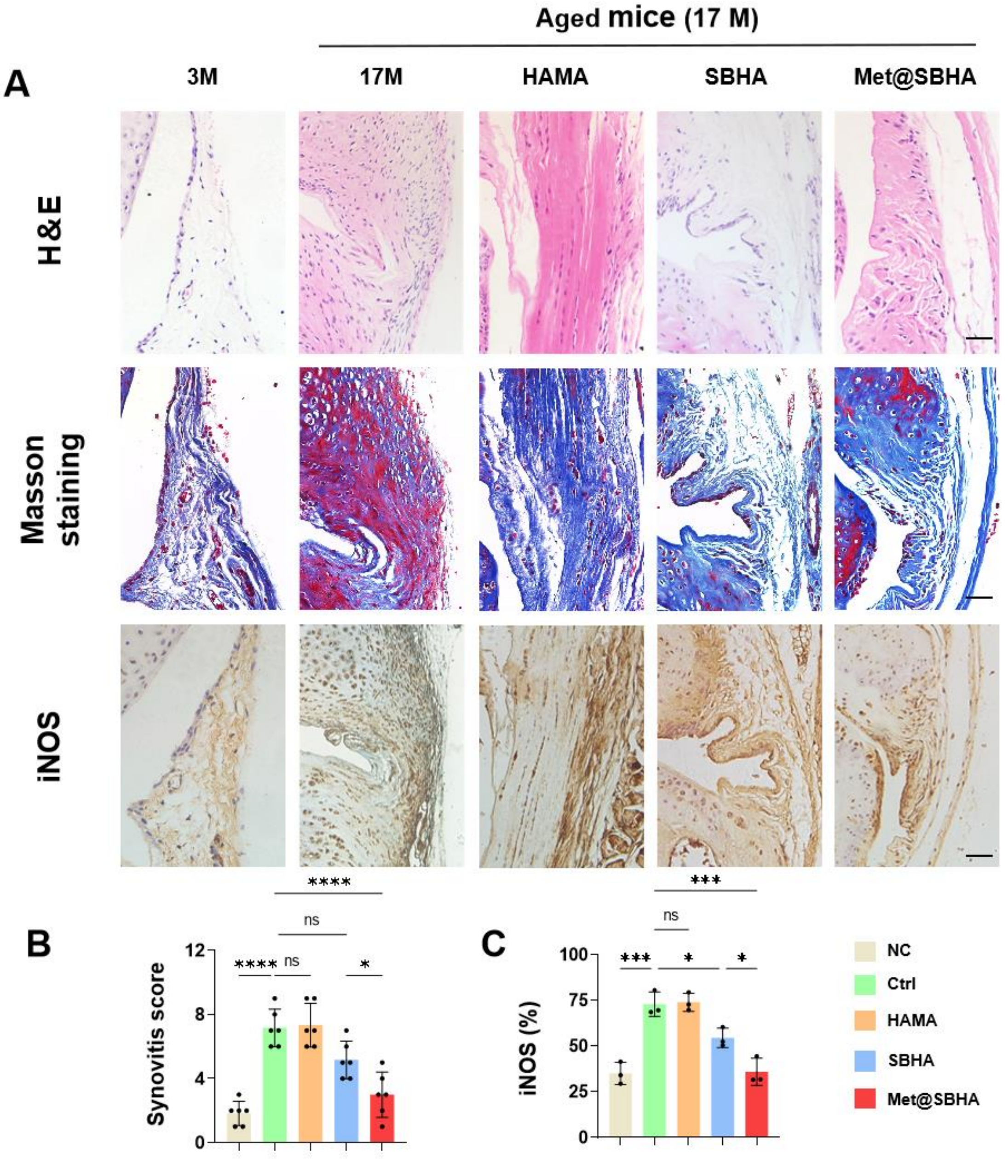

Figure S14. Histological staining and scoring of synovial tissue. **A**) HE staining, Masson staining and iNOS immunohistochemical staining of synovium. **B**) Total synovitis score for each synovial group according to the degree of proliferation of synovial cells, blood vessels and fibrous tissue. **C**) Statistical analysis of iNOS expression levels in synovial membranes. Scale bar 100 μm. Number of repetitions of all experiments $n \geq 3$. Data are presented as the mean ± SD. (*, $P < 0.05$; **, $P < 0.01$; ***, $P < 0.001$ and ****, $P <0.0001$)

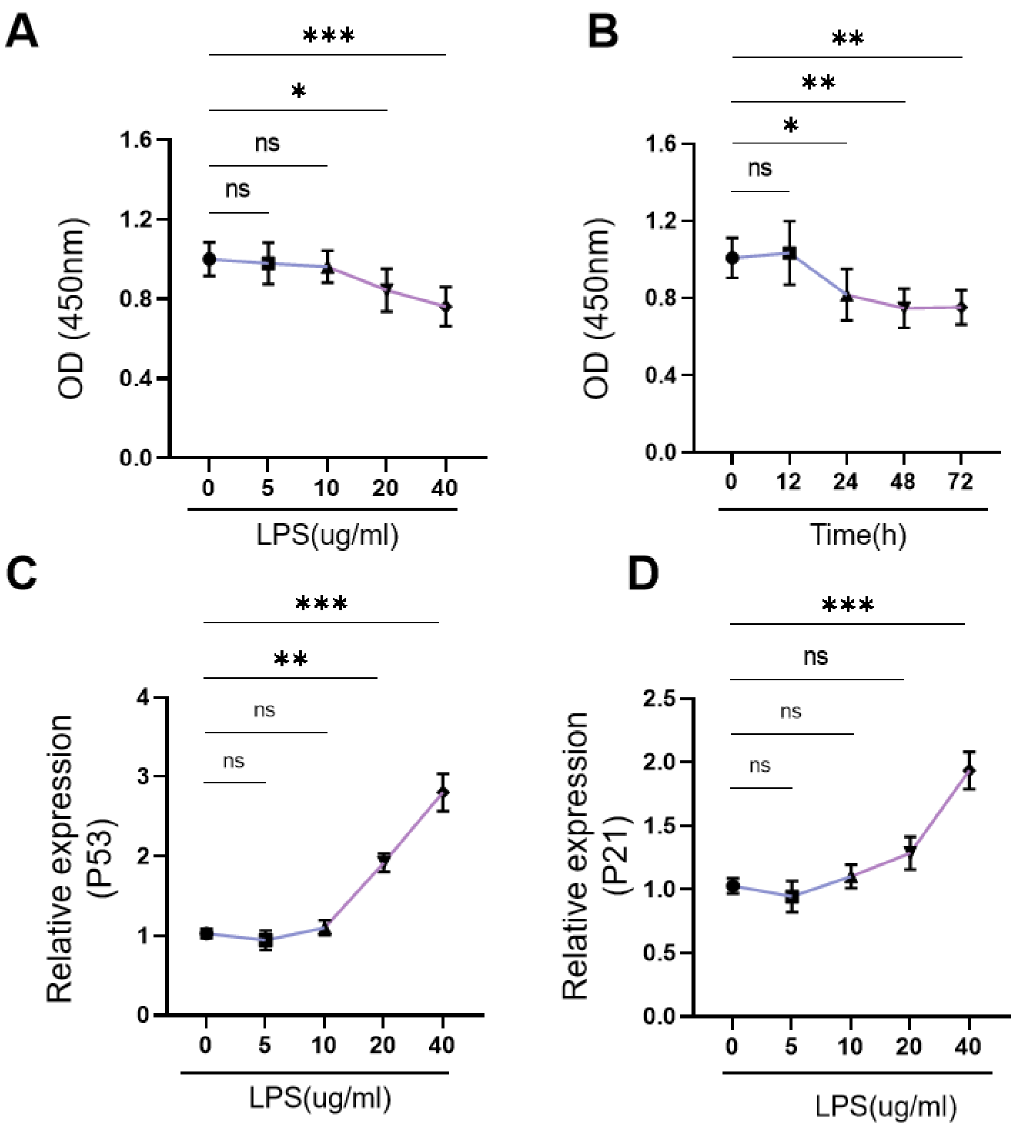

Figure S15. Construction of cell senescence models. **A**) Concentration gradient LPS for cell viability. **B**) Time gradient LPS assay for cell viability. **C-D)** RT-PCR detection relative mRNA expression levels of P53 and P21 under concentration gradient LPS treatment. Number of repetitions of all experiments n ≥ 3. Data are presented as the mean ± SD. (*, P < 0.05; **, P < 0.01; ***, P < 0.001 and ****, P <0.0001)

	Rq (nm)	Ra (nm)	DMT modulus (MPa)	Force-displacement curves			
				maximum peak pressure (nN)	adsorption force (nN)	deformation (nm)	Young's modulus (kPa)
**HAMA**	64.9	85.5	749	40.02	10.25	16.43	7.09
**SBHA**	46.1	62.5	822	40.28	26.24	22.14	9.59

Table S1. Specific values of mechanical properties of HAMA and SBHA microspheres detected by AFM

Gene	Target	Mean Binding energy	Run
NOS1	6png	-5.653	1
NOS1	6png	-5.628	2
NOS1	6png	-5.569	3
NOS1	6png	-5.564	4
NOS1	6png	-5.555	5
NOS1	6png	-5.328	6
NOS1	6png	-4.991	7
NOS1	6png	-4.779	8
NOS1	6png	-4.744	9
NOS2	6jwn	-5.022	1
NOS2	6jwn	-4.938	2
NOS2	6jwn	-4.464	3
NOS2	6jwn	-4.443	4
NOS2	6jwn	-4.415	5
NOS2	6jwn	-4.382	6
NOS2	6jwn	-4.324	7
NOS2	6jwn	-4.279	8
NOS2	6jwn	-4.206	9
NOS3	6nh8	-5.606	1
NOS3	6nh8	-5.363	2
NOS3	6nh8	-5.231	3
NOS3	6nh8	-4.712	4
NOS3	6nh8	-4.606	5
NOS3	6nh8	-4.596	6
NOS3	6nh8	-4.166	7
NOS3	6nh8	-4.15	8
NOS3	6nh8	-4.118	9
EGFR	8a27	-5.502	1
EGFR	8a27	-5.121	2
EGFR	8a27	-5.06	3
EGFR	8a27	-4.998	4
EGFR	8a27	-4.811	5
EGFR	8a27	-4.66	6
EGFR	8a27	-4.35	7
EGFR	8a27	-4.343	8
EGFR	8a27	-4.272	9
PDE5A	1xor	-5.569	1
PDE5A	1xor	-5.455	2
PDE5A	1xor	-5.447	3
PDE5A	1xor	-5.169	4
PDE5A	1xor	-5.059	5
PDE5A	1xor	-4.824	6

PDE5A	1xor	-4.801	7
PDE5A	1xor	-4.755	8
PDE5A	1xor	-4.614	9
PDE5A	1xoz	-5.777	1
PDE5A	1xoz	-5.406	2
PDE5A	1xoz	-5.196	3
PDE5A	1xoz	-4.822	4
PDE5A	1xoz	-4.781	5
PDE5A	1xoz	-4.541	6
PDE5A	1xoz	-4.117	7
PDE5A	1xoz	-3.963	8
PDE5A	1xoz	-3.928	9
IDO1	5ek4	-5.231	1
IDO1	5ek4	-4.643	2
IDO1	5ek4	-4.593	3
IDO1	5ek4	-4.533	4
IDO1	5ek4	-4.463	5
IDO1	5ek4	-4.389	6
IDO1	5ek4	-4.261	7
IDO1	5ek4	-4.133	8
IDO1	5ek4	-4.082	9
IDO1	5etw	-4.769	1
IDO1	5etw	-4.699	2
IDO1	5etw	-4.684	3
IDO1	5etw	-4.537	4
IDO1	5etw	-4.255	5
IDO1	5etw	-4.136	6
IDO1	5etw	-3.95	7
IDO1	5etw	-3.857	8
IDO1	5etw	-3.695	9
F2	5iuq	-3.369	1
F2	5iuq	-3.292	2
F2	5iuq	-3.221	3
F2	5iuq	-3.102	4
F2	5iuq	-3.054	5
F2	5iuq	-3.031	6
F2	5iuq	-2.893	7
F2	5iuq	-2.884	8
F2	5iuq	-2.613	9
F9	5jb9	-5.124	1
F9	5jb9	-4.916	2
F9	5jb9	-4.829	3
F9	5jb9	-4.78	4
F9	5jb9	-4.754	5

F9	5jb9	-4.462	6
F9	5jb9	-4.045	7
F9	5jb9	-4.003	8
F9	5jb9	-3.92	9
F9	5jba	-4.682	1
F9	5jba	-4.645	2
F9	5jba	-4.513	3
F9	5jba	-4.115	4
F9	5jba	-4.064	5
F9	5jba	-4.01	6
F9	5jba	-3.997	7
F9	5jba	-3.919	8
F9	5jba	-3.517	9
ESR2	7xvy	-5.208	1
ESR2	7xvy	-4.635	2
ESR2	7xvy	-4.513	3
ESR2	7xvy	-4.494	4
ESR2	7xvy	-4.308	5
ESR2	7xvy	-4.172	6
ESR2	7xvy	-4.169	7
ESR2	7xvy	-4.105	8
ESR2	7xvy	-3.957	9

Table S2. Docking scores were obtained for twelve compounds

Gene	primer sequence	
P53	Forward Sequence	CTGGTTAGTCCTGAGACGAGG
	Reverse Sequence	AGATGCAGCCAAACACAGCAC
P21\CDKN1A	Forward Sequence	TCGCTGTCTTGCACTCTGGTGTGT
	Reverse Sequence	CCAATCTGCGCTTGGAGTGATAG
MMP13	Forward Sequence	GATGACCTGTCTGAGGAAGACC
	Reverse Sequence	GCATTTCTCGGAGCCTGTCAAC
COL2A1	Forward Sequence	GCTGGTGAAGAAGGCAAACGAG
	Reverse Sequence	CCATCTTGACCTGGGAATCCAC
IL-1β	Forward Sequence	TGGACCTTCCAGGATGAGGACA
	Reverse Sequence	GTTCATCTCGGAGCCTGTAGTG
IL-6	Forward Sequence	TACCACTTCACAAGTCGGAGAGGGC
	Reverse Sequence	CTGCAAGTGCATCATCGTTGTTC
GAPDH	Forward Sequence	CATCACTGCCACCCAGAAGACTG
	Reverse Sequence	ATGCCAGTGAGCTTCCCGTTCAG

Table S3. Specific primers employed for mRNA amplification
